# The Space of Equidistant Phylogenetic Cactuses

**DOI:** 10.1007/s00026-023-00656-0

**Published:** 2023-06-09

**Authors:** Katharina T. Huber, Vincent Moulton, Megan Owen, Andreas Spillner, Katherine St. John

**Affiliations:** 1 School of Computing Sciences, University of East Anglia, Norwich, NR4 7TJ UK; 2 Department of Mathematics, Lehman College, CUNY, New York, NY 10468 USA; 3 Merseburg University of Applied Sciences, 06217 Merseburg, Germany; 4 Department of Computer Science, Hunter College, CUNY, New York, NY 10065 USA

**Keywords:** Phylogenetic network, Network space, Combinatorial encoding, CAT(0)-metric space, 05C90, 06A06, 52B70, 92D15

## Abstract

An *equidistant* *X*-*cactus* is a type of rooted, arc-weighted, directed acyclic graph with leaf set *X*, that is used in biology to represent the evolutionary history of a set $X$ of species. In this paper, we introduce and investigate the space of equidistant *X*-cactuses. This space contains, as a subset, the space of ultrametric trees on *X* that was introduced by Gavryushkin and Drummond. We show that equidistant-cactus space is a CAT(0)-metric space which implies, for example, that there are unique geodesic paths between points. As a key step to proving this, we present a combinatorial result concerning *ranked* rooted *X*-cactuses. In particular, we show that such graphs can be encoded in terms of a pairwise compatibility condition arising from a poset of collections of pairs of subsets of $X$ that satisfy certain set-theoretic properties. As a corollary, we also obtain an encoding of ranked, rooted *X*-trees in terms of partitions of *X*, which provides an alternative proof that the space of ultrametric trees on *X* is CAT(0). We expect that our results will provide the basis for novel ways to perform statistical analyses on collections of equidistant *X*-cactuses, as well as new directions for defining and understanding spaces of more general, arc-weighted phylogenetic networks.

## Introduction

Currently, there is great interest in developing theory and techniques to understand and construct (rooted) *phylogenetic networks*. Generally speaking, for a set of species, such a network consists of a rooted, directed acyclic graph and a bijective map from the species to the set of sinks of the graph (in case the graph is a tree, the network is called a (rooted) *phylogenetic tree*). Phylogenetic networks are important as they can be used to represent the evolutionary history of species that cross with one another (through evolutionary processes such as hybridization and recombination). To date, much of the research on phylogenetic networks has focused on understanding the structure of special types of networks and ways to build them (see [34] for a recent overview of the area). More recently, however, as the theory for phylogenetic networks has developed, there has been growing interest in understanding how to equip collections of phylogenetic networks with suitable metrics, giving rise to so-called *network spaces*. As has been demonstrated for the intensively studied spaces of phylogenetic trees (cf. e.g. [8, 18], and the review [32]), or *tree-spaces*, this point of view is valuable as it provides insights into statistical approaches to analyze and systematically compare networks.

Network spaces essentially come in two types: discrete and continuous. In *discrete* spaces, the elements of the space are distinct, non-isomorphic networks, and a metric is commonly given by defining the distance between two networks to be the length of a minimal sequence of local network operations that converts one network into the other. In *continuous* spaces, the arcs in the networks have non-negative, real-valued lengths and one network can be converted into the other by shrinking or lengthening arcs in a continuous manner. To date, nearly all results on network spaces have concerned discrete spaces (see, for example, [9, 17, 24], for related results on discrete spaces of unrooted networks see e.g. [23]). Indeed, to the best of our knowledge, very few results have been presented on continuous network spaces except for the recently introduced spaces of (unrooted) circular split networks^1^ [16]. This is probably in part because the study of phylogenetic networks with arc lengths is somewhat less developed than the study of those without.

**Fig. 1:**
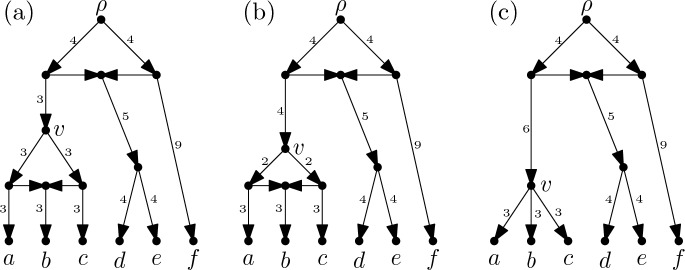
**a** An $X$-cactus for $X = \{a,b,c,d,e,f\}$ with root $\rho$ that is equidistant since every directed path from $\rho$ to a sink has the same length, namely $13$. All incoming arcs at vertices with indegree 2 have length 0 and are drawn horizontally. **b** The rooted $X$-cactus obtained by lengthening the incoming arc and shrinking the outgoing arcs at vertex $v$ by 1. **c** The rooted $X$-cactus obtained by continuing the lengthening and shrinking of the arcs at vertex $v$ until both outgoing arcs have length 0, contracting the cycle below $v$ completely

In this paper, we introduce a new continuous space of phylogenetic networks that can be regarded as a generalization of the $\tau$-*space* of ultrametric trees that was introduced in [18]. For a set *X* of species, our network space ${\mathfrak {N}}(X)$ is comprised of *equidistant* $X$-*cactuses* (see Fig. 1a for an example of such a network). A rooted *X*-cactus is essentially a rooted phylogenetic network in which no two distinct cycles in the underlying graph have an arc in common. Note that if all vertices of a rooted $X$-cactus have indegree at most 1 the network is just a *rooted phylogenetic* $X$-*tree*. The extensively studied class of (rooted) *level-1 networks* (see e.g. [29]) also provides examples of rooted *X*-cactuses. Assigning a non-negative real-valued length to each of the arcs in a rooted phylogenetic network, then such a network ${\mathcal {N}}$ is called *equidistant* if, for any fixed vertex $v$ of ${\mathcal {N}}$, all directed paths from $v$ to any sink of ${\mathcal {N}}$ have the same length. Algorithms for constructing equidistant phylogenetic networks have been studied in, e.g., [10] and [13].

Following one of the common approaches used to construct tree-spaces, we define equidistant-cactus space ${\mathfrak {N}}(X)$ in terms of an *orthant space* (see e.g. [25]). Basically, an orthant space is a collection of real orthants that are glued together along their boundaries and that is equipped with the metric induced by using the Euclidean metric within each orthant. That is, the distance between two points in the same orthant is the Euclidean distance between these points, and the distance between two points in different orthants is the length of a shortest path, or *geodesic path*, between these points. The length of such a path is computed by summing the Euclidean lengths of the restrictions of the path to each orthant. In particular, each pair of points in ${\mathfrak {N}}(X)$ represents two equidistant $X$-cactuses, and moving along a geodesic path between the points continuously converts one $X$-cactus into the other by shrinking and lengthening arcs (see Fig. 1b, c), which may also result in a change of the length of the paths from the root to the sinks. Note that the points of $\tau$-space correspond bijectively to equidistant *X*-trees and that it can be constructed by gluing together orthants indexed by *ranked phylogenetic trees*. We take a similar approach to define ${\mathfrak {N}}(X)$, indexing orthants instead by *ranked* $X$-*cactuses*, in which a ranking of the vertices that respects the direction of the arcs in the rooted $X$-cactus is given. We remark that ranked phylogenetic networks have been recently introduced and that research has focused on counting and enumerating certain classes of such networks (see e.g. [7, 12] and the references therein).

A critical aspect that influenced our construction of ${\mathfrak {N}}(X)$ was that—as has been shown for $\tau$-space [18]—we wanted it to be a *CAT(0)-metric space*. Being CAT(0) is an important geometrical property that has been exploited in various applications within phylogenetics and beyond (see e.g. [3, 14]). A space being CAT(0) immediately implies that there is a *unique* geodesic path between any two points, a property that underpins many useful computations that can be performed for tree- and orthant-spaces. More specifically, approximations of the median as well as of the Fréchet mean and variance can be computed in complete CAT(0)-metric spaces, which include CAT(0)-orthant spaces [4, 25]; a central limit theorem holds for CAT(0)-orthant spaces [5]; and methods for computing confidence sets [37] and an analogue of partial principal component analysis [26, 27] can be directly extended from the unrooted tree space presented in [8] to CAT(0)-orthant spaces. Most of this paper is devoted to proving a crucial combinatorial result concerning rooted *X*-cactuses (Theorem 2) which implies, via a classical result of Gromov for orthant spaces, that ${\mathfrak {N}}(X)$ is CAT(0). In passing, we remark that the space of networks described in [16] is not a CAT(0)-metric space.

The rest of this paper is structured as follows. In Sect. 2, we formally define rooted $X$-cactuses as well as some related concepts. In Sect. 3, we then introduce rankings of rooted $X$-cactuses and equidistant *X*-cactuses, which are both defined in terms of so-called time-stamp functions. As well as characterizing when a rooted $X$-cactus admits a ranking of its vertices that is consistent with the direction of its arcs, we make an important observation concerning ranked *X*-cactuses (Lemma 2), which implies that the maximal chains in a certain poset mentioned in the next paragraph all have the same length, i. e.  $|X |-1$. In Sect. 4, we use the simpler case of equidistant $X$-trees to outline our approach for the construction of a network space that is CAT(0), including a new proof that $\tau$-space is CAT(0).

In Sect. 5, we describe how ranked $X$-cactuses give rise to *set pair systems* as defined in [22] and present the properties that characterize set pair systems that arise from ranked $X$-cactuses. We also define a binary relation on general set pair systems, and in Sect. 6, we establish that this relation yields a bounded graded poset on the set pair systems that arise from ranked $X$-cactuses. In Sect. 7, we establish our main combinatorial result (Theorem 2), namely that chains in this poset encode ranked $X$-cactuses. In simpler terms, this can be regarded as a “pairwise compatibility” result for set pair systems, which is analogous to the well-known *Splits Equivalence Theorem* for unrooted phylogenetic trees (see e.g. [30, Theorem 3.1.4]). Using our encoding for ranked $X$-cactuses, in Sect. 8, we construct the space ${\mathfrak {N}}(X)$ of equidistant *X*-cactuses and show that it is a CAT(0)-metric space. We conclude in Sect. 9 by mentioning some directions for future work.

## Preliminaries

In this section, we define rooted *X*-cactuses and some related concepts that we use later. We begin by recalling some standard concepts from graph theory. A *directed graph* $N=(V,A)$ consists of a finite non-empty set $V$ and a subset $A \subseteq V \times V$. The elements of $V$ and $A$ are referred to as *vertices* and *arcs* of $N$, respectively. A directed graph $N$ is *acyclic* if there is no directed cycle in $N$. Moreover, a directed acyclic graph (DAG) $N$ is *rooted* if there exists a vertex $\rho \in V$ with indegree $0$, called the *root* of $N$, such that for every $u \in V$ there is a directed path from $\rho$ to $u$. In a rooted DAG, a *leaf* is a vertex with outdegree 0, an *internal vertex* is a vertex with outdegree at least 1, a *tree vertex* is a vertex with indegree at most 1 and a *reticulation vertex* is a vertex with indegree at least 2. Note that, by definition, the root of a rooted DAG is a tree vertex. Moreover, in a rooted DAG $N$, we call a vertex $v$ a *child* of a vertex $u$ and, similarly, $u$ a *parent* of $v$ if $(u,v)$ is an arc of $N$. The set of children of a vertex $u$ is denoted by $ch(u)$. A *reticulation cycle* $\{P,P'\}$ in a rooted DAG consists of two distinct directed paths $P$ and $P'$ such that $P$ and $P'$ have the same start vertex and the same end vertex but no other vertices in common.

Let $X$ be a finite non-empty set. A *rooted* $X$-*cactus* ${\mathcal {N}} = (N,\varphi )$ is a rooted DAG $N=(V,A)$ together with a map $\varphi : X \rightarrow V$ such that

(RC1): all vertices of $N$ have indegree at most 2,
(RC2): no two distinct reticulation cycles in $N$ have an arc in common, and
(RC3): the image $\varphi (X)$ contains all leaves and all tree vertices of $N$ with outdegree $1$ of $N$.

**Fig. 2:**
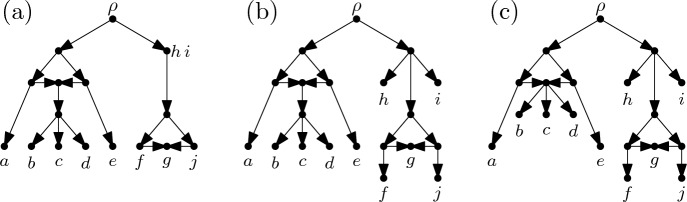
**a** A rooted $X$-cactus ${\mathcal {N}}$ for $X=\{a,b,c,\dots ,j\}$. **b** The rooted, phylogenetic $X$-cactus $\widehat{{\mathcal {N}}}$. **c** The rooted, compressed, phylogenetic $X$-cactus $\widehat{{\mathcal {N}}}^*$

In Fig. 2a, we give an example of a rooted $X$-cactus. We remark that if $|X |= 1$ a rooted $X$-cactus consists of a single vertex only. For better readability, we will often refer to the vertices and arcs of $N$ as the vertices and arcs of ${\mathcal {N}}$. A rooted $X$-cactus ${\mathcal {N}}$ is *phylogenetic*^2^ if $\varphi$ is a bijection between $X$ and the set of leaves of ${\mathcal {N}}$. Note that a rooted phylogenetic $X$-cactus may contain leaves that are reticulation vertices. A rooted $X$-cactus is *binary* if it is phylogenetic, all leaves of ${\mathcal {N}}$ are tree vertices, the root has outdegree 2 and every other internal vertex has either indegree 1 and outdegree 2 or indegree 2 and outdegree 1. A rooted $X$-cactus ${\mathcal {N}}$ is *compressed* if $\varphi (X)$ also contains all reticulation vertices with outdegree 1 (see [34, p. 251] for the concept of compression in more general phylogenetic networks). Rooted, compressed, phylogenetic $X$-cactuses as defined here correspond to 1-nested phylogenetic networks as defined in [22]. Note that a rooted, binary $X$-cactus that contains at least one reticulation vertex cannot be compressed. A rooted $X$-cactus without any reticulation vertices is called a *rooted* $X$-*tree*. Note that rooted $X$-trees as defined here are in one-to-one correspondence with the rooted $X$-trees as defined in [30] where the root is required to have outdegree 1.

In Sect. 7, we will need to associate with every rooted $X$-cactus ${\mathcal {N}} = ((V,A),\varphi )$ a rooted, phylogenetic $X$-cactus $\widehat{{\mathcal {N}}} = (({\widehat{V}},{\widehat{A}}),{\widehat{\varphi }})$ as follows: For every $x \in X$ such that $\varphi (x)$ is not a leaf of ${\mathcal {N}}$ or such that there exists some $y \in X - \{x\}$ with $\varphi (y) = \varphi (x)$ we add a new vertex $u$ to $V$, add the arc $(\varphi (x),u)$ to $A$, and put ${\widehat{\varphi }}(x) = u$. For all other $x \in X$ we put ${\widehat{\varphi }}(x) = \varphi (x)$. The resulting set of vertices and arcs, respectively, are denoted by ${\widehat{V}}$ and ${\widehat{A}}$ (see Fig. 2b). In addition, we associate with the resulting rooted, phylogenetic $X$-cactus $\widehat{{\mathcal {N}}}$ the rooted, compressed, phylogenetic $X$-cactus $\widehat{{\mathcal {N}}}^* = (({\widehat{V}}^*,{\widehat{A}}^*),{\widehat{\varphi }}^*)$ obtained by contracting all arcs $(u,v)$ where $u$ has outdegree 1 (see Fig. 2c).

## Rankings, Time-Stamp Functions and Equidistant *X*-Cactuses

In this section, we consider rankings of the vertices of rooted *X*-cactuses, which are an important part of defining equidistant-cactus space. It is convenient to start with the more general concept of time-stamp functions, which also naturally leads to the definition of equidistant *X*-cactuses. A *time-stamp function* on the vertices in a rooted $X$-cactus ${\mathcal {N}} = ((V,A),\varphi )$ is a map $t: V \rightarrow {\mathbb {R}}_{\ge 0}$ such that

(TS1): $t(v)=0$ for all $v \in \varphi (X)$,
(TS2): $t(u) > t(v)$ for all arcs $(u,v)$ of ${\mathcal {N}}$ with $v$ not a reticulation vertex, and
(TS3): $t(v) = t(p_1) = t(p_2)$ for all reticulation vertices $v$ of ${\mathcal {N}}$ and its two parents $p_1$ and $p_2$.

**Fig. 3:**
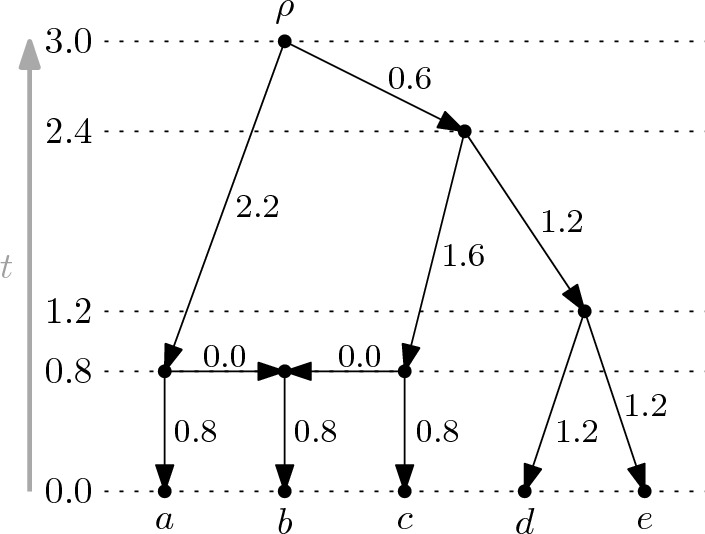
A rooted $X$-cactus ${\mathcal {N}}$ on $X=\{a,b,c,d,e\}$ with a time-stamp function $t$ on its vertices. For all vertices $v$ the value $t(v)$ is given by the real number to the left of the horizontal line through $v$. In addition, for each arc of ${\mathcal {N}}$, the length of the arc induced by $t$ is given

An example of a time-stamp function on the vertices of a rooted $X$-cactus is given in Fig. 3. Integer-valued time-stamp functions are also known as *temporal labelings* (see e.g. [6]). We call a rooted $X$-cactus ${\mathcal {N}}$ *temporal* if there exists a time-stamp function on the vertices of ${\mathcal {N}}$. Note that not every rooted $X$-cactus is temporal (for example, the rooted $X$-cactus in Fig. 2a is not temporal because $\varphi (X)$ contains an internal vertex that is not a parent of a reticulation vertex). The following lemma characterizes rooted $X$-cactuses that are temporal (see also [6, Theorem 3] for a characterization that applies to general rooted phylogenetic networks).

### Lemma 1

A rooted $X$-cactus ${\mathcal {N}} = ((V,A),\varphi )$ is temporal if and only if for all vertices $u \in V$ the following properties hold:

- If $u \in \varphi (X)$ then either $u$ is a leaf or a parent of a reticulation vertex that is a leaf.
- If $u$ has outdegree at least 2 then $u$ is not the parent of a reticulation vertex that is a leaf.
- If $u$ is the parent of a reticulation vertex $v$ in a reticulation cycle $\{P,P'\}$ then neither of the directed paths $P$, $P'$ consists of the single arc $(u,v)$.

### Proof

First assume that ${\mathcal {N}}$ is temporal. Consider a time-stamp function $t$ on the vertices of ${\mathcal {N}}$. Assuming that ${\mathcal {N}}$ contains a vertex $u$ that violates one of (a)–(c) immediately yields a contradiction because then $t$ would violate at least one of (TS1)–(TS3).

Now assume that (a)–(c) hold for all vertices of ${\mathcal {N}}$. We construct a time-stamp function $t$ on the vertices of ${\mathcal {N}}$ by first putting $t(v) = 0$ for all $v \in \varphi (X)$. In view of (a) and (b), this does not violate (TS1)–(TS3).

Next, consider an internal vertex $u$ that is not a reticulation vertex and also not the parent of a reticulation vertex. Assume that all children $w$ of $u$ have been assigned time-stamps $t(w)$. Then we put $t(u) = 1 + \max _{w \in ch(u)} t(w)$. Since ${\mathcal {N}}$ is acyclic this does not violate (TS1)–(TS3).

Finally, consider an internal vertex $u$ that is a reticulation vertex. Let $p_1$ and $p_2$ denote the two parents of $u$ and assume that all vertices $w$ in

$$\begin{aligned}M = (ch(u) \cup ch(p_1) \cup ch(p_2)) - \{u\}\end{aligned}$$

have been assigned time-stamps $t(w)$. Then, we put $t(u) = t(p_1) = t(p_2) = 1 + \max _{w \in M} t(w)$. Since ${\mathcal {N}}$ is acyclic and in view of (c) this does not violate (TS1)–(TS3).

Thus, our inductive construction yields a map $t: V \rightarrow {\mathbb {R}}_{\ge 0}$ for which (TS1)–(TS3) hold. $\square$

As indicated in Fig. 3, a time-stamp function $t$ on the vertices of a rooted $X$-cactus ${\mathcal {N}} = ((V,A),\varphi )$ induces non-negative lengths on the arcs of ${\mathcal {N}}$ by putting the length of arc $(u,v)$ to be $t(u)-t(v)$. With these arc lengths, all directed paths from a fixed vertex $u$ to a vertex $w \in \varphi (X)$ have the same length, namely $t(u)$. In view of this, we call an ordered pair $({\mathcal {N}},t)$ consisting of a rooted, temporal $X$-cactus ${\mathcal {N}}$ and a time-stamp function $t$ on the vertices of ${\mathcal {N}}$ an *equidistant* $X$-cactus. Thus, an equidistant $X$-cactus can be thought of as a rooted, temporal $X$-cactus with specific arc lengths assigned, whereas a rooted, temporal $X$-cactus does not have any specific arc lengths assigned.

**Fig. 4:**
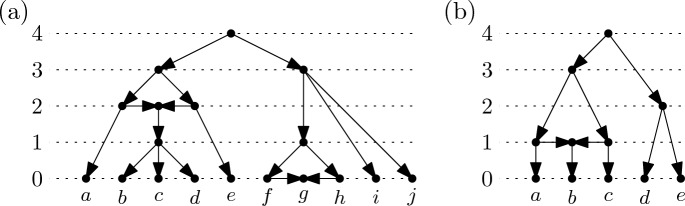
**a** A ranking of size 4 of a rooted $X$-cactus with $X=\{a,b,c,\dots ,j\}$. Vertices of the same rank are drawn on the same horizontal line. **b** A ranking of a rooted, binary $X$-cactus with $X=\{a,b,c,d,e\}$. The ranking has size 4 which is the maximum size over all rooted, temporal $X$-cactuses with $|X |= 5$

We conclude this section by shedding some more light on the combinatorial structure of rooted, temporal $X$-cactuses. The *size* $\sigma (t)$ of a time-stamp function $t$ on the vertices of a rooted, temporal $X$-cactus ${\mathcal {N}} = ((V,A),\varphi )$ is $|t(V) |- 1$. A *ranking* of a rooted, temporal $X$-cactus ${\mathcal {N}} = ((V,A),\varphi )$ is a time-stamp function $r$ on the vertices of ${\mathcal {N}}$ with $r(V) = \{0,1,2,\dots ,\sigma (r)\}$. See Fig. 4a for an example. Note that rankings as defined here are a particular type of temporal labeling and are more general than the rankings considered in [7]. The value $r(v)$ assigned to vertex $v$ by the ranking $r$ will also be referred to as the *rank* of vertex $v$ if the ranking referred to is clear from the context. A *ranked* $X$-cactus $({\mathcal {N}},r)$ consists of a rooted, temporal $X$-cactus ${\mathcal {N}}$ and a ranking $r$ of the vertices of ${\mathcal {N}}$. The following lemma gives tight bounds on the size of rankings of rooted, temporal $X$-cactuses (see Fig. 4b for an example). For its proof, we will use the fact that any rooted binary $X$-cactus can be transformed into a rooted binary $X$-tree by deleting, for every reticulation vertex $v$, one of the arcs $(p,v)$ from a parent $p$ of $v$ to $v$ and then suppressing the two internal vertices $v$ and $p$.

### Lemma 2

Let $({\mathcal {N}},r)$ be a ranked $X$-cactus. Then, we have $0 \le \sigma (r) \le |X |- 1$. Moreover,

- $\sigma (r) = 0$ if and only if ${\mathcal {N}}$ consists of a single vertex.
- $\sigma (r) = |X |-1$ if and only if ${\mathcal {N}}$ is a rooted, binary $X$-cactus and $r(u) \ne r(v)$ for all distinct vertices $u$ and $v$ unless $u$ and $v$ are both leaves of ${\mathcal {N}}$, $u$ is a parent of a reticulation vertex $v$, or $u$ and $v$ are parents of the same reticulation vertex.

### Proof

By definition, $\sigma (r) \ge 0$. Moreover, if the size of the ranking $r$ is precisely 0 then ${\mathcal {N}}$ must consist of a single leaf $v$ with $r(v) = 0$ and all elements of $X$ are mapped by $\varphi$ to $v$.

To establish the upper bound, let $i$ and $k$ denote the number of internal and reticulation vertices, respectively, of the ranked $X$-cactus $({\mathcal {N}},r)$. By definition, $\sigma (r) \le (i - 2k)$. Note that, for fixed $X$, this expression can only be maximum if ${\mathcal {N}}$ is a rooted, binary $X$-cactus, because otherwise we can always increase $i$ without increasing $k$. Hence, it suffices to show that for all rooted, binary $X$-cactuses we have $i - 2k = |X |- 1$. Since, as described above, we can transform any such $X$-cactus into a rooted binary $X$-tree, we immediately obtain this equation as a consequence of the well-known fact that a rooted binary $X$-tree has $|X |- 1$ internal vertices (see e.g. [30, Sec. 2.1]). $\square$

## Equidistant *X*-Trees and $\tau$-Space

In this section, we shall briefly recall the concept of an orthant space (see e.g. [25, Sec. 6]) and related concepts. To illustrate the basic idea for constructing our orthant space of equidistant cactuses, we also consider the simpler case of equidistant trees (often called ultrametric trees) and explain how the $\tau$-space of ultrametric trees mentioned in the introduction arises as an orthant space. This also yields an alternative proof to the one presented in [18] for the fact that $\tau$-space is a CAT(0)-metric space.

### Orthant Spaces

An ordered pair $(M,{\mathcal {F}})$ consisting of a family ${\mathcal {F}}$ of non-empty subsets of a finite non-empty set $M$ is called an *abstract simplicial complex* if $A \in {\mathcal {F}}$ implies that all non-empty subsets of $A$ are also contained in ${\mathcal {F}}$. An abstract simplicial complex is a *flag complex* if, for all non-empty subsets $A \subseteq M$ such that all two-element subsets of $A$ are contained in ${\mathcal {F}}$, we have $A \in {\mathcal {F}}$. For every map $\omega : M \rightarrow {\mathbb {R}}_{\ge 0}$, we put $\text {supp}(\omega ) = \{x \in M: \omega (x) > 0\}$. The *orthant space* associated with the abstract simplicial complex $(M,{\mathcal {F}})$ is

$$\begin{aligned}{\mathfrak {M}}_{(M,{\mathcal {F}})} = \left\{ \omega \in {\mathbb {R}}_{\ge 0}^M: \text {supp}(\omega ) \in {\mathcal {F}} \cup \{\emptyset \}\right\} .\end{aligned}$$

A *metric* $D$ on a non-empty set $B$ is a map $D: B \times B \rightarrow {\mathbb {R}}_{\ge 0}$ such that

- $D(x,y) = 0$ if and only if $x=y$,
- $$D(x,y) = D(y,x)$$, and
- $$D(x,z) \le D(x,y) + D(y,z)$$

hold for all $x,y,z \in B$. The ordered pair $(B,D)$ is called a *metric space* and the elements of $B$ are called the *points* of the metric space. A metric $D_{(M,{\mathcal {F}})}$ on the orthant space ${\mathfrak {M}}_{(M,{\mathcal {F}})}$ associated with the abstract simplicial complex $(M,{\mathcal {F}})$ can be constructed as follows. For every $A \in {\mathcal {F}}$, the set

$$\begin{aligned}{\mathfrak {O}}(A) = \{\omega \in {\mathfrak {M}}_{(M,{\mathcal {F}})}: \text {supp}(\omega ) \subseteq A\}\end{aligned}$$

is called an *orthant* of ${\mathfrak {M}}_{(M,{\mathcal {F}})}$. For all $\omega ,\omega ' \in {\mathfrak {M}}_{(M,{\mathcal {F}})}$ such that there exists an orthant ${\mathfrak {O}}$ of ${\mathfrak {M}}_{(M,{\mathcal {F}})}$ with $\{\omega ,\omega '\} \subseteq {\mathfrak {O}}$ we put

$$\begin{aligned}D_{(M,{\mathcal {F}})}(\omega ,\omega ') = \sqrt{\sum _{x \in M} (\omega (x) - \omega '(x))^2}.\end{aligned}$$

Then, for all $\omega ,\omega ' \in {\mathfrak {M}}_{(M,{\mathcal {F}})}$ such that there is no orthant ${\mathfrak {O}}$ of ${\mathfrak {M}}_{(M,{\mathcal {F}})}$ that contains both $\omega$ and $\omega '$ we consider finite *segmented paths* from $\omega$ to $\omega '$. These are sequences $\omega _0,\omega _1,\omega _2,\dots ,\omega _k$ of elements in ${\mathfrak {M}}_{(M,{\mathcal {F}})}$ such that $\omega =\omega _0$, $\omega '=\omega _k$ and, for all $i \in \{1,2,\dots ,k\}$, there exists some orthant ${\mathfrak {O}}_i$ of ${\mathfrak {M}}_{(M,{\mathcal {F}})}$ that contains both $\omega _{i-1}$ and $\omega _i$. The *length* of such a segmented path is $\sum _{i=1}^k D_{(M,{\mathcal {F}})}(\omega _{i-1},\omega _{i})$. Note that at least one such segmented path always exists in view of the fact that all orthants of ${\mathfrak {M}}_{(M,{\mathcal {F}})}$ contain the point $\omega$ with $\text {supp}(\omega ) = \emptyset$, called the *origin* of ${\mathfrak {M}}_{(M,{\mathcal {F}})}$. We define $D_{(M,{\mathcal {F}})}(\omega ,\omega ')$ to be the infimum of the length of all segmented paths from $\omega$ to $\omega '$. It is known (see [25, Sec. 6]) that this construction yields a metric space $({\mathfrak {M}}_{(M,{\mathcal {F}})},D_{(M,{\mathcal {F}})})$.

Next, we describe a useful property that the metric space $({\mathfrak {M}}_{(M,{\mathcal {F}})},D_{(M,{\mathcal {F}})})$ may have. A *geodesic path* between the points $p$ and $q$ in a metric space $(B,D)$ is a map $\gamma : [0,\ell ] \rightarrow B$, for some $\ell \ge 0$, with $\gamma (0)=p$, $\gamma (\ell ) = q$ and $D(\gamma (t_1),\gamma (t_2)) = \vert t_1 - t_2 |$ for all $t_1,t_2 \in [0,\ell ]$. A metric space $(B,D)$ is *geodesic* if there exists a geodesic path between $p$ and $q$ for all $p,q \in B$. A geodesic metric space $(B,D)$ is a $\text {CAT}(0)$-metric space if and only if (see e.g. [11, p. 163])

$$\begin{aligned}(D(p,q))^2 + (D(p,r))^2 \ge 2(D(m,p))^2 + (D(q,r))^2/2\end{aligned}$$

holds for all $p,q,r \in B$ and all $m \in B$ with $D(q,m) = D(r,m) = D(q,r)/2$. $\text {CAT}(0)$-metric spaces arise in many applications (see e.g. [3]). They have the important property that geodesic paths are unique [11, Proposition 1.4, p. 160]. It follows from a result in [19] that the orthant space $({\mathfrak {M}}_{(M,{\mathcal {F}})},D_{(M,{\mathcal {F}})})$ is a $\text {CAT}(0)$-metric space if and only if ${\mathcal {F}}$ is a flag complex (see also [25, Proposition 6.14]). Furthermore, geodesic paths can be computed in polynomial time in $\text {CAT}(0)$-orthant spaces [25, Corollary 6.19].

### $\tau$-Space Revisited

To describe how the $\tau$-space of ultrametric trees arises as an orthant space, we start with a suitably defined abstract simplicial complex. A *partition* of $X$ is a set ${\mathcal {P}}$ of non-empty and pairwise disjoint subsets of $X$ with $X = \bigcup _{A \in {\mathcal {P}}} A$. We denote the set of all partitions of $X$ by ${\mathfrak {B}}(X)$ and define a binary relation $\sqsubseteq$ on ${\mathfrak {B}}(X)$ by putting ${\mathcal {P}}_1 \sqsubseteq {\mathcal {P}}_2$ if for all $A_1 \in {\mathcal {P}}_1$ there exists some $A_2 \in {\mathcal {P}}_2$ with $A_1 \subseteq A_2$. Intuitively, this means that the partition ${\mathcal {P}}_1$ refines the partition ${\mathcal {P}}_2$. It is well-known that $\sqsubseteq$ is a partial ordering. Note that the partial ordering $\sqsubseteq$ is induced by the partial ordering $\subseteq$ on the subsets of $X$.

**Fig. 5:**
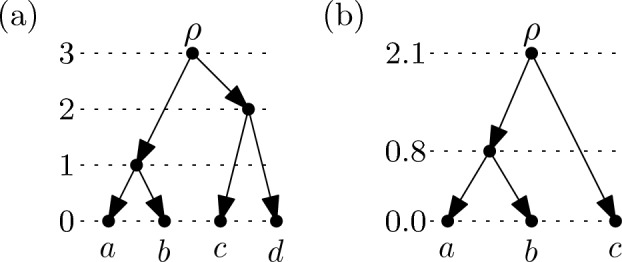
**a** A ranked $X$-tree with $X=\{a,b,c,d\}$. Any cut along one of the dotted horizontal lines yields a partition of $X$ (for example, the dotted line labeled with 1 yields the partition $\{\{a,b\},\{c\},\{d\}\}$). **b** An equidistant $X$-tree with $X=\{a,b,c\}$

Every ranked $X$-tree with a ranking of size $\sigma$ gives rise to a sequence

$$\begin{aligned}{\mathcal {P}}_0 \sqsubseteq {\mathcal {P}}_1 \sqsubseteq \dots \sqsubseteq {\mathcal {P}}_{\sigma } = \{X\}\end{aligned}$$

of partitions of $X$. In Fig. 5a, we depict a rooted $X$-tree with a ranking of size $\sigma = 3$ that gives rise to the sequence

$$\begin{aligned}\{\{a\},\{b\},\{c\},\{d\}\} \sqsubseteq \{\{a,b\},\{c\},\{d\}\} \sqsubseteq \{\{a,b\},\{c,d\}\} \sqsubseteq \{\{a,b,c,d\}\}\end{aligned}$$

(see also Sect. 5.1 where we formally define how the partitions arise more generally for ranked $X$-cactuses). The crucial fact is that this sequence encodes the ranked *X*-tree. More formally, as we shall prove as a consequence of our results for general ranked $X$-cactuses in Corollary 2, we have:

#### Theorem 1

There is a one-to-one correspondence between (isomorphism classes of) ranked $X$-trees and subsets of ${\mathfrak {B}}(X)$ that contain $\{X\}$ and that consist of partitions of $X$ which are pairwise comparable with respect to the partial ordering $\sqsubseteq$.

To obtain $\tau$-space as an orthant space, we consider the abstract simplicial complex $({\mathfrak {B}}^{\circ }(X),{\mathcal {F}}(\sqsubseteq ))$ with ${\mathfrak {B}}^{\circ }(X) = {\mathfrak {B}}(X) - \{X\}$ and ${\mathcal {F}}(\sqsubseteq )$ containing all non-empty subsets of ${\mathfrak {B}}^{\circ }(X)$ whose elements are pairwise comparable with respect to $\sqsubseteq$. It follows immediately that $({\mathfrak {B}}^{\circ }(X),{\mathcal {F}}(\sqsubseteq ))$ is a flag complex. Note that, more generally, we can associate an abstract simplicial complex that is a flag complex to any partial ordering in an analogous way; for this reason such a complex is known as an *order complex* (see e.g. [36, p. 248]).

**Fig. 6:**
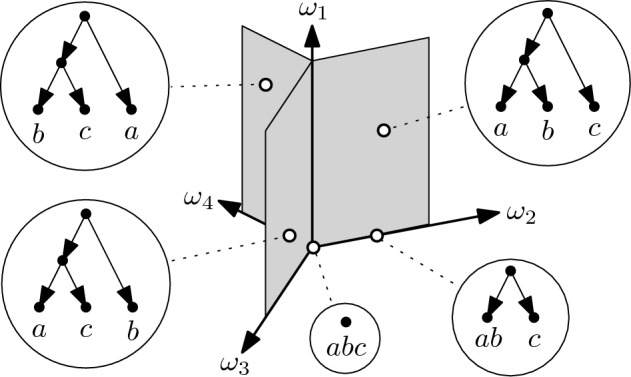
The orthant space ${\mathfrak {M}}_{({\mathfrak {B}}^{\circ }(X),{\mathcal {F}}(\sqsubseteq ))}$ for $X=\{a,b,c\}$. By construction, each axis represents a partition of $X$ distinct from $\{X\}$. The axes labeled $\omega _1$ and $\omega _2$, for example, represent the partitions $\{\{a\},\{b\},\{c\}\}$ and $\{\{a,b\},\{c\}\}$, respectively. The three two-dimensional orthants are drawn shaded. All points in the interior of these two-dimensional orthants correspond to the same isomorphism class of binary ranked $X$-trees. The rankings for them are not shown because they are unique. Points on the axes correspond to non-binary ranked $X$-trees. The origin corresponds to the ranked $X$-tree that consists of a single vertex

**Fig. 7:**
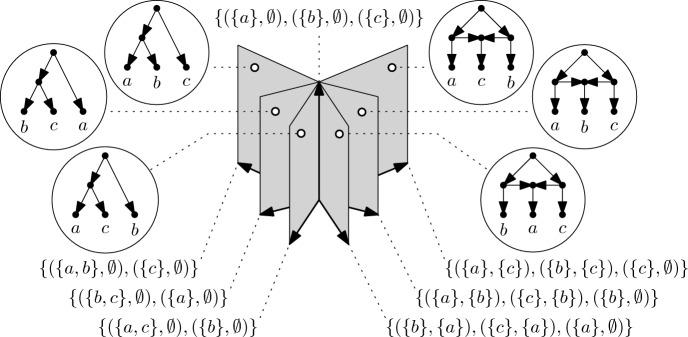
The structure of ${\mathfrak {N}}(X)$ for $X=\{a,b,c\}$. The six two-dimensional orthants are drawn shaded. Each of these two-dimensional orthants corresponds to an isomorphism class of binary ranked $X$-cactuses. Each axis corresponds to the indicated polestar system on $X$

In Fig. 6, we illustrate the orthant space ${\mathfrak {M}}_{({\mathfrak {B}}^{\circ }(X),{\mathcal {F}}(\sqsubseteq ))}$ of equidistant *X*-trees for $X=\{a,b,c\}$ (see Fig. 7 for an analogous drawing of the resulting orthant space of equidistant $X$-cactuses). Note that, by construction, the coordinates of a point in any orthant are obtained as differences between consecutive time stamps in the equidistant *X*-tree that corresponds to the point. The equidistant $X$-tree in Fig. 5b, for example, corresponds to the point $(\omega _1,\omega _2,\omega _3,\omega _4) = (0.8,1.3,0,0)$. More generally, it follows by Theorem 1 that the elements in ${\mathfrak {M}}_{({\mathfrak {B}}^{\circ }(X),{\mathcal {F}}(\sqsubseteq ))}$ are in one-to-one correspondence with equidistant *X*-trees. Moreover, since $({\mathfrak {B}}^{\circ }(X),{\mathcal {F}}(\sqsubseteq ))$ is a flag complex, it follows, as mentioned in Sect. 4.1, that the resulting metric space $({\mathfrak {M}}_{({\mathfrak {B}}^{\circ }(X),{\mathcal {F}}(\sqsubseteq ))},D_{({\mathfrak {B}}^{\circ }(X),{\mathcal {F}}(\sqsubseteq ))})$ is CAT(0). We remark that, by construction, ${\mathfrak {M}}_{({\mathfrak {B}}^{\circ }(X),{\mathcal {F}}(\sqsubseteq ))}$ is precisely $\tau$-space, and so we obtain an alternative proof to the one presented in [18] that $\tau$-space is a CAT(0)-metric space.

Before proceeding, we note that in [21] the problem of when a partition of $X$ is compatible with a rooted phylogenetic $X$-tree is studied. This includes, as a special case, the situation where the vertices of the tree can be ranked in such a way that the partition is among those associated with the resulting ranked $X$-tree. In addition, in [2] a space, called the *Bergman fan* of the matroid of the complete graph with vertex set $X$ is studied. This space is a polyhedral fan and its points are also in one-to-one correspondence with equidistant $X$-trees. Although not an orthant space, its cones are in one-to-one correspondence with the orthants of ${\mathfrak {M}}_{({\mathfrak {B}}^{\circ }(X),{\mathcal {F}}(\sqsubseteq ))}$.

## An Encoding for Ranked *X*-Cactuses

To help the reader navigate the remaining sections of this paper, we now briefly summarize how we shall construct the equidistant-cactus space ${\mathfrak {N}}(X)$ by applying an analogue of the process described in Sect. 4.2.

We shall begin by introducing the concept of a *polestar system* on the set *X*, which is a collection of ordered pairs of subsets of $X$, or set pair system for short, with certain properties. As we shall see in Sect. 5.2, polestar systems can be associated to ranked *X*-cactuses in a similar way how partitions can be associated to ranked *X*-trees. We shall also define a binary relation $\preceq$ on general set pair systems, and, in Sect. 6, we will show that $\preceq$ yields a partial ordering on the set ${\mathfrak {P}}(X)$ of polestar systems on *X*. In Sect. 7, we then prove an analogue of Theorem 1, namely, we show that ranked *X*-cactuses are in one-to-one correspondence with subsets of ${\mathfrak {P}}(X)$ that contain the maximum element relative to the ordering $\preceq$ and that are pairwise comparable with respect to $\preceq$. In other words, we obtain an encoding of ranked $X$-cactuses in terms of certain collections of polestar systems. In Sect. 9, we conclude by constructing the network space ${\mathfrak {N}}(X)$ as the orthant space associated to the order complex of the poset $({\mathfrak {P}}(X),\preceq )$.

### Set Pair Systems

Before introducing polestar systems, we recall the concept of a set pair system introduced in [22]. To this end, we say that a vertex *u* in a rooted DAG $N$ is a *descendant* of a vertex *v* if there exists a directed path from the root of $N$ to $u$ that contains $v$. A descendant $u$ of $v$ is a *strict descendant* if every directed path from the root to *u* contains *v*. Otherwise $u$ is called a *non-strict descendant* of $v$. Now, given a rooted $X$-cactus ${\mathcal {N}} = ((V,A),\varphi )$ and a vertex $u \in V$, let $C(u)$ be the set of those $x \in X$ with $\varphi (x)$ a descendant of *u*, *S*(*u*) the set of those $x \in X$ with $\varphi (x)$ a strict descendant of *u* and *H*(*u*) the set of those $x \in X$ with $\varphi (x)$ a non-strict descendant of *u* in *X*. For every vertex $u$ of ${\mathcal {N}}$, we call $(S(u),H(u))$ the *set pair* associated to $u$ and put

$$\begin{aligned}{\mathcal {S}}({\mathcal {N}}) = \{(S(u),H(u)): u \in V\}.\end{aligned}$$

For later reference, we state some immediate consequences of the definition of the set pairs in ${\mathcal {S}}({\mathcal {N}})$ for a rooted $X$-cactus ${\mathcal {N}}$ (see also [22] where these properties have been considered in the context of the slightly more restrictive 1-nested phylogenetic networks):

(SH1): For all vertices $u$ of ${\mathcal {N}}$, we have $S(u) \cap H(u) = \emptyset$, $S(u) \cup H(u) = C(u)$ and *S*(*u*) is always non-empty while *H*(*u*) may be empty.
(SH2): If $(S(u),H(u)) = (S(v),H(v))$ for two distinct vertices $u$ and $v$ of ${\mathcal {N}}$ then one of these vertices, say $u$, is a reticulation vertex with outdegree 1 and $v$ is the single child of $u$. Note that this situation cannot occur if ${\mathcal {N}}$ is compressed.
(SH3): Let $C$ be the set of vertices in a reticulation cycle of ${\mathcal {N}}$ where $u$ and $v$ are the common start and end vertex, respectively, of the two directed paths that form the reticulation cycle. Then we have $H(w) = S(v)$ if $w \in C - \{u,v\}$ and, for all other vertices $w'$ of ${\mathcal {N}}$, we have $H(w') \ne S(v)$.

Now, given a ranked $X$-cactus $({\mathcal {N}}=((V,A),\varphi ),r)$ we collect, for every $i \in \{0,1,2,\dots ,\sigma (r)\}$, in ${\mathcal {S}}_i({\mathcal {N}})$ first those set pairs from ${\mathcal {S}}({\mathcal {N}})$ that correspond to vertices of rank at most $i$ and whose parents (if any) have rank strictly larger than $i$. We then add some further set pairs that essentially help to keep track of the fact that some of the vertices involved are in a reticulation cycle. More formally, we define $V_i$ to be the set that consists of all vertices $u \in V$ with $r(u) \le i$ and $r(p) > i$ for all parents $p$ of $u$. Note that, in view of (TS3), $V_i$ does not contain any reticulation vertices. Thus, all $u \in V_i$ have at most one parent. Then, we put

$$\begin{aligned}{\mathcal {S}}_i({\mathcal {N}}) = \{(S(u),H(u)): u \in V_i\} \cup \{(H(u),\emptyset ): u \in V_i, \ H(u) \ne \emptyset \}.\end{aligned}$$

Note that we always have ${\mathcal {S}}_{\sigma (r)} = \{(X,\emptyset )\}$. For the rooted $X$-cactus ${\mathcal {N}}$ in Fig. 4a, for example, we obtain:

$$\begin{aligned} {\mathcal {S}}_4({\mathcal {N}})&= \{(\{a,b,c,d,e,f,g,h,i,j\},\emptyset )\}\\ {\mathcal {S}}_3({\mathcal {N}})&= \{(\{a,b,c,d,e\},\emptyset ),(\{f,g,h,i,j\},\emptyset )\}\\ {\mathcal {S}}_2({\mathcal {N}})&= \{(\{a\},\{b,c,d\}),(\{b,c,d\},\emptyset ),(\{e\},\{b,c,d\}), (\{f,g,h\},\emptyset ),(\{i\},\emptyset ),(\{j\},\emptyset )\}\\ {\mathcal {S}}_1({\mathcal {N}})&= \{(\{a\},\emptyset ),(\{b,c,d\},\emptyset ),(\{e\},\emptyset ), (\{f,g,h\},\emptyset ),(\{i\},\emptyset ),(\{j\},\emptyset )\}\\ {\mathcal {S}}_0({\mathcal {N}})&= \{(\{x\},\emptyset ): x \in \{a,b,c,d,e,g,i,j\}\} \cup \{(\{f\},\{g\}),(\{h\},\{g\})\} \end{aligned}$$

A collection of ordered pairs (*S*, *H*) of subsets of $X$ such that $S \ne \emptyset$ and $S \cap H = \emptyset$ is called a *set pair system* on $X$. Note that, by construction, the sets ${\mathcal {S}}({\mathcal {N}})$ and ${\mathcal {S}}_i({\mathcal {N}})$, $0 \le i \le \sigma (r)$, associated with a ranked $X$-cactus $({\mathcal {N}},r)$ are non-empty set-pair systems.

It is shown in [22] that, for any set pair system ${\mathcal {S}}$ on $X$, we obtain a partial ordering $\le$ on the set pairs in ${\mathcal {S}}$ by putting $(S_1,H_1) \le (S_2,H_2)$ if either $(S_1,H_1) = (S_2,H_2)$ or $(S_1,H_1) \ne (S_2,H_2)$ and one the following holds:

- $$S_1 \cup H_1 \subseteq S_2$$
- $$S_1 \cup H_1 \subseteq H_2$$
- $$S_1 \subsetneq S_2$$ and $$H_1 = H_2 \ne \emptyset $$

We write $(S_1,H_1) < (S_2,H_2)$ if $(S_1,H_1) \le (S_2,H_2)$ and the set pairs $(S_1,H_1)$ and $(S_2,H_2)$ are distinct. The partial ordering $\le$ on set pairs was defined in such a way that we have $(S(u),H(u)) \le (S(v),H(v))$ for two vertices $u$ and $v$ in a rooted $X$-cactus if and only if $u$ is a descendant of $v$ (see the proof of Theorem 5 in [22]).

We use the partial ordering $\le$ on set pairs to define a binary relation $\preceq$ on set pair systems. More precisely, for set pair systems ${\mathcal {S}}_1$ and ${\mathcal {S}}_2$ on $X$ we put ${\mathcal {S}}_1 \preceq {\mathcal {S}}_2$ if

(SP1): for all $(S_1,H_1) \in {\mathcal {S}}_1$, there exists some $(S_2,H_2) \in {\mathcal {S}}_2$ with $(S_1,H_1) \le (S_2,H_2)$, and
(SP2): for all $(S_2,H_2) \in {\mathcal {S}}_2$ with $H_2 \ne \emptyset$, if there exists some $(S_1,H_1) \in {\mathcal {S}}_1$ with $H_1 = H_2$, then there exists such a $(S_1,H_1)$ with $(S_1,H_1) \le (S_2,H_2)$.

Again, we write ${\mathcal {S}}_1 \prec {\mathcal {S}}_2$ if ${\mathcal {S}}_1 \preceq {\mathcal {S}}_2$ and ${\mathcal {S}}_1 \ne {\mathcal {S}}_2$. We remark that (SP1) captures the basic idea from Sect. 4.2 that the partial ordering $\le$ on set pairs induces a suitable binary relation on set pair systems (in analogy to how the partial ordering $\subseteq$ induced the binary relation $\sqsubseteq$). (SP2) is an additional technical requirement that will be crucial in our encoding of ranked $X$-cactuses.

The relation $\preceq$ is, in general, not a partial ordering on the set pair systems on a fixed set $X$, because it might neither be antisymmetric nor transitive. For the set pair systems associated with a ranked $X$-cactus, however, the following holds.

#### Lemma 3

Let $({\mathcal {N}},r)$ be a ranked $X$-cactus. Then we have ${\mathcal {S}}_i({\mathcal {N}}) \prec {\mathcal {S}}_j({\mathcal {N}})$ for all $0 \le i < j \le \sigma (r)$ and ${\mathcal {S}}_{\sigma (r)}({\mathcal {N}}) = \{(X,\emptyset )\}$.

#### Proof

As noted earlier in this section, ${\mathcal {S}}_{\sigma (r)}({\mathcal {N}}) = \{(X,\emptyset )\}$ follows immediately from the definition of the set pair system ${\mathcal {S}}_{\sigma (r)}({\mathcal {N}})$. Consider $0 \le i < j \le \sigma (r)$. We first show that ${\mathcal {S}}_i({\mathcal {N}}) \preceq {\mathcal {S}}_j({\mathcal {N}})$. Therefore, consider $(S,H) \in {\mathcal {S}}_i({\mathcal {N}})$. By definition of ${\mathcal {S}}_i({\mathcal {N}})$, there must exist a vertex $v$ in ${\mathcal {N}}$ with $r(v) \le i$, $r(p) > i$ for all parents $p$ of $v$, and either $(S,H) = (S(v),H(v))$ or $(S,H) = (H(v),\emptyset )$. Consider a directed path from the root of ${\mathcal {N}}$ to $v$. On this path, there must exist a vertex $u$ with $r(u) \le j$ and $r(p) > j$ for all parents $p$ of $u$. This implies that $(S(u),H(u)) \in {\mathcal {S}}_j({\mathcal {N}})$. Moreover, in view of the fact that $u$ lies on a directed path from the root of ${\mathcal {N}}$ to $v$, we must have $(S,H) \le (S(v),H(v)) \le (S(u),H(u))$, as required by (SP1).

To establish that also (SP2) is satisfied for ${\mathcal {S}}_i({\mathcal {N}})$ and ${\mathcal {S}}_j({\mathcal {N}})$, consider $(S,H) \in {\mathcal {S}}_j({\mathcal {N}})$ with $H \ne \emptyset$. By definition of ${\mathcal {S}}_j({\mathcal {N}})$, there must exist a vertex $u$ in ${\mathcal {N}}$ with $(S,H) = (S(u),H(u))$, $r(u) \le j$ and $r(p) > j$ for all parents $p$ of $u$. Now, if there exists some $(S',H') \in {\mathcal {S}}_i({\mathcal {N}})$ with $H'=H$, then there exists some vertex $v$ in ${\mathcal {N}}$ with $(S',H') = (S',H) = (S(v),H(v))$, $r(v) \le i$ and $r(p) > i$ for all parents $p$ of $v$. This implies that $u$ and $v$ must be vertices in the same reticulation cycle of ${\mathcal {N}}$. Moreover, we can choose $v$ such that $v$ is a descendant of $u$, implying that $(S',H') = (S(v),H(v)) \le (S(u),H(u)) = (S,H)$, as required.

It remains to show that ${\mathcal {S}}_i({\mathcal {N}}) \ne {\mathcal {S}}_j({\mathcal {N}})$. By the definition of a ranked $X$-cactus, there must exist a vertex $u \in V$ with $r(u) = j$. Without loss of generality, we may assume that $u$ is not a reticulation vertex. If $(S(u),H(u)) \not \in {\mathcal {S}}_i({\mathcal {N}})$ we are done. Therefore, assume for a contradiction that $(S(u),H(u)) \in {\mathcal {S}}_i({\mathcal {N}})$. In view of $i < j$ we have $u \not \in V_i$. Thus, there exists some $v \ne u$ in $V_i$ such that either (i) $H(v) \ne \emptyset$ and $(S(u),H(u)) = (H(v),\emptyset )$ or (ii) $(S(u),H(u)) = (S(v),H(v))$. If Case (i) holds then, in view of (SH3), $v$ must be a vertex in a reticulation cycle with end vertex $u'$ and $(S(u'),H(u')) = (H(v),\emptyset ) = (S(u),H(u))$. Since $u$ is not a reticulation vertex, it follows, by (SH2), that $u$ is the single child of $u'$. Consequently, $i=r(v) > r(u) = j$, a contradiction. Similarly, if Case (ii) holds then, again by (SH2), it follows that $u$ is a reticulation vertex and $v$ is the single child of $u$, a contradiction. $\square$

### Polestar Systems

A set pair system ${\mathcal {S}}$ on $X$ is *partition-like* if

(PL1): ${\mathcal {P}}({\mathcal {S}}) = \{S: (S,H) \in {\mathcal {S}}\}$ is a partition of $X$,
(PL2): for all $(S,H),(S',H') \in {\mathcal {S}}$ with $(S,H) \ne (S',H')$ we have $S \ne S'$, and
(PL3): for all $(S,H) \in {\mathcal {S}}$ with $H \ne \emptyset$ we have $(H,\emptyset ) \in {\mathcal {S}}$ and there exists precisely one $(S',H') \in {\mathcal {S}}$ with $(S',H') \ne (S,H)$ and $H=H'$.

A partition-like set pair system is called a *polestar system*, for short. In addition, we define ${\mathcal {H}}({\mathcal {S}}) = \{H: (S,H) \in {\mathcal {S}}, H \ne \emptyset \}$. Note that (PL2) implies that $|{\mathcal {S}} |= |{\mathcal {P}}({\mathcal {S}}) |$.

#### Lemma 4

Let $({\mathcal {N}},r)$ be a ranked $X$-cactus. Then, ${\mathcal {S}}_i({\mathcal {N}})$ is a polestar system for all $0 \le i \le \sigma (r)$.

#### Proof

Fix some $i \in \{0,1,\dots ,\sigma (r)\}$ and consider two distinct vertices $u_1, u_2 \in V_i$. Put $(S_k,H_k) = (S(u_k),H(u_k))$, $k \in \{1,2\}$. Recall from the definition of the set $V_i$ that both $u_1$ and $u_2$ have rank at most $i$ while the ranks of their parents are strictly larger than $i$. Thus, up to switching the roles of $u_1$ and $u_2$, one of the following must hold:

- Neither of $u_1$ and $u_2$ is a descendant of the other and there is no reticulation cycle in ${\mathcal {N}}$ that contains both $u_1$ and $u_2$. Consequently, $(S(u_1) \cup H(u_1)) \cap (S(u_2) \cup H(u_2)) = \emptyset$. Thus, the sets $S(u_1)$, $H(u_1)$, $S(u_2)$ and $H(u_2)$ are pairwise disjoint.
- Both $u_1$ and $u_2$ are contained in the same reticulation cycle in ${\mathcal {N}}$ but neither is a descendant of the other. Consequently, $H(u_1) = H(u_2) = H \ne \emptyset$ and the sets $S(u_1)$, $S(u_2)$ and $H$ are pairwise disjoint.

It follows from this case analysis that (PL1) and (PL2) hold for ${\mathcal {S}}_i({\mathcal {N}})$.

To see that also (PL3) holds, consider a set pair $(S,H) \in {\mathcal {S}}_i({\mathcal {N}})$ with $H \ne \emptyset$. By the definition of ${\mathcal {S}}_i({\mathcal {N}})$, there must exist a vertex $u$ in ${\mathcal {N}}$ with $(S(u),H(u)) = (S,H)$ such that $r(u) \le i$ and $r(p) > i$ for all parents $p$ of $u$. In view of $H(u) = H \ne \emptyset$, vertex $u$ must be contained in a reticulation cycle ${\mathcal {C}}$ but cannot be the common start or the common end vertex of the two directed paths that form ${\mathcal {C}}$. Note that ${\mathcal {C}}$ contains a unique vertex $v \ne u$ with $r(v) \le i$ and $r(p) > i$ for all parents $p$ of $v$. Moreover, $v$ cannot be the common start or the common end vertex of the two directed paths that form ${\mathcal {C}}$. Since $u$ and $v$ are both contained in ${\mathcal {C}}$, we have $H(u) = H(v) = H$. Moreover, by (SH3), there are no other vertices $w$ in ${\mathcal {N}}$ with $H(w) = H$, $r(w) \le i$ and $r(p) > i$ for all parents $p$ of $w$. Finally, by construction, we also have $(H,\emptyset ) = (H(u),\emptyset ) \in {\mathcal {S}}_i({\mathcal {N}})$. $\square$

We denote by ${\mathfrak {P}}(X)$ the set of polestar systems on the set $X$. Note that, even for the set pair systems in ${\mathfrak {P}}(X)$, (SP1) in the definition of the binary relation $\preceq$ does not imply (SP2), as can be seen from the set pair systems

$$\begin{aligned} {\mathcal {S}}_1&= \{(\{a\},\{b\}),(\{b\},\emptyset ),(\{c\},\{b\}),(\{d\},\emptyset )\} \ \text {and}\\ {\mathcal {S}}_2&= \{(\{a,c\},\{b\}),(\{b\},\emptyset ),(\{d\},\{b\})\} \end{aligned}$$

on $X=\{a,b,c,d\}$ which satisfy (PL1)–(PL3) and (SP1) but not (SP2).

We conclude this section with two technical lemmas stating some properties of the relations $\le$ and $\preceq$ that will be used in Sects. 6 and 7. In particular, Lemma 5 establishes that, up to a specific exception, distinct set pairs within a single polestar system are incomparable with respect to the partial ordering $\le$ and the binary relations $\le$ and $\preceq$ are consistent. In our encoding of ranked $X$-cactuses, this exception corresponds to the set pairs associated with reticulation vertices.

#### Lemma 5

Let ${\mathcal {S}}_1,{\mathcal {S}}_2 \in {\mathfrak {P}}(X)$ with ${\mathcal {S}}_1 \preceq {\mathcal {S}}_2$. Then, for all $(S_1,H_1) \in {\mathcal {S}}_1$ and $(S_2,H_2) \in {\mathcal {S}}_2$, $(S_2,H_2) < (S_1,H_1)$ implies $(S_2,H_2) \in {\mathcal {S}}_1$, $H_2 = \emptyset$ and $H_1 = S_2$.

#### Proof

First, consider the case ${\mathcal {S}}_1 = {\mathcal {S}}_2 = {\mathcal {S}}$. Let $(S_1,H_1), (S_2,H_2) \in {\mathcal {S}}$ with $(S_2,H_2) < (S_1,H_1)$. Assume for a contradiction that $H_2 \ne \emptyset$. Then, in view of (PL1)–(PL3), none of $S_2 \cup H_2 \subseteq S_1$, $S_2 \cup H_2 \subseteq H_1$ and $S_2 \subsetneq S_1$ can hold, in contradiction to $(S_2,H_2) < (S_1,H_1)$. Thus, we must have $H_2 = \emptyset$. Consequently, $S_2 \subseteq H_1$, and, therefore, $S_2=H_1$, as required.

Next, consider the case ${\mathcal {S}}_1 \prec {\mathcal {S}}_2$. Let $(S_1,H_1) \in {\mathcal {S}}_1$ and $(S_2,H_2) \in {\mathcal {S}}_2$ with $(S_2,H_2) < (S_1,H_1)$. In view of ${\mathcal {S}}_1 \prec {\mathcal {S}}_2$, there must exist some $(S_2',H_2') \in {\mathcal {S}}_2$ with $(S_1,H_1) \le (S_2',H_2')$. By the transitivity of $\le$, we obtain $(S_2,H_2) < (S_2',H_2')$. In view of the first case considered in this proof, this implies $S_2 = H_2'$ and $H_2 = \emptyset$. Thus, by the definition of a set pair, we have $S_2 \cap S_2' = \emptyset$. Moreover, $(S_2,H_2) < (S_1,H_1) \le (S_2',H_2')$ simplifies to $(S_2,\emptyset ) < (S_1,H_1) \le (S_2',S_2)$. In view of the definition of $\le$, the latter can only hold if $S_2 = H_1 \ne \emptyset$. By (PL3), this implies $(H_1,\emptyset ) = (S_2,H_2) \in {\mathcal {S}}_1$, as required. $\square$

#### Lemma 6

Let ${\mathcal {S}}_1,{\mathcal {S}}_2 \in {\mathfrak {P}}(X)$ with ${\mathcal {S}}_1 \prec {\mathcal {S}}_2$. Then,

$$\begin{aligned}1 \le |{\mathcal {P}}({\mathcal {S}}_2) |- |{\mathcal {H}}({\mathcal {S}}_2) |< |{\mathcal {P}}({\mathcal {S}}_1) |- \vert {\mathcal {H}}({\mathcal {S}}_1) |\le |X |.\end{aligned}$$

If $(|{\mathcal {P}}({\mathcal {S}}_1) |- |{\mathcal {H}}({\mathcal {S}}_1)|) - (|{\mathcal {P}}({\mathcal {S}}_2) |- |{\mathcal {H}}({\mathcal {S}}_2) |) \ge 2$ then there exists ${\mathcal {S}}_3 \in {\mathfrak {P}}(X)$ with ${\mathcal {S}}_1 \prec {\mathcal {S}}_3 \prec {\mathcal {S}}_2$.

#### Proof

In view of (PL1), we have $1 \le |{\mathcal {P}}({\mathcal {S}}) |\le |X |$ for all ${\mathcal {S}} \in {\mathfrak {P}}(X)$. Moreover, in view of (PL3), we have $|{\mathcal {P}}({\mathcal {S}}) |\ge 3 |{\mathcal {H}}({\mathcal {S}}) |$. This implies $1 \le |{\mathcal {P}}({\mathcal {S}}) |- |{\mathcal {H}}({\mathcal {S}}) |\le |X |$.

Next consider ${\mathcal {S}}_1,{\mathcal {S}}_2 \in {\mathfrak {P}}(X)$ with ${\mathcal {S}}_1 \prec {\mathcal {S}}_2$. We first show that, for all $S' \in {\mathcal {P}}({\mathcal {S}}_1)$, there exists a unique $S'' \in {\mathcal {P}}({\mathcal {S}}_2)$ with $S' \subseteq S''$. In view of (PL2), there exists a unique set pair $(S_1,H_1) \in {\mathcal {S}}_1$ with $S' = S_1$ and, in view of ${\mathcal {S}}_1 \prec {\mathcal {S}}_2$, there must exist a set pair $(S_2,H_2) \in {\mathcal {S}}_2$ with $(S_1,H_1) \le (S_2,H_2)$. Therefore, by the definition of $\le$, one of the following must hold:

- $S_1 \cup H_1 \subseteq S_2$. Then, we put $S''=S_2$.
- $S_1 \cup H_1 \subseteq H_2$. This implies $H_2 \ne \emptyset$ and thus, by (PL3), $H_2 \in {\mathcal {P}}({\mathcal {S}}_2)$. We put $S'' = H_2$.
- $S_1 \subsetneq S_2$ and $H_1 = H_2 \ne \emptyset$. Then, we put $S'' = S_2$.

In each case, we have $S' \subseteq S''$ for some $S'' \in {\mathcal {P}}({\mathcal {S}}_2)$ and, in view of (PL1), $S''$ is unique, as claimed. This implies that we obtain a map $q: {\mathcal {S}}_1 \rightarrow {\mathcal {S}}_2$ by assigning to each $(S',H') \in {\mathcal {S}}_1$ the unique $(S'',H'') \in {\mathcal {S}}_2$ with $S' \subseteq S''$. In particular, we have $|{\mathcal {P}}({\mathcal {S}}_1)|\ge |{\mathcal {P}}({\mathcal {S}}_2) |$.

To establish $|{\mathcal {P}}({\mathcal {S}}_2) |- |{\mathcal {H}}({\mathcal {S}}_2) |< |{\mathcal {P}}({\mathcal {S}}_1) |- |{\mathcal {H}}({\mathcal {S}}_1)|$, put $k = |{\mathcal {P}}({\mathcal {S}}_1)|- |{\mathcal {P}}({\mathcal {S}}_2)|$. Let $\ell _1$ denote the number of $H' \in {\mathcal {H}}({\mathcal {S}}_1)$ with $H' \not \in {\mathcal {H}}({\mathcal {S}}_2)$. Note that, in view of (PL3), for each such $H'$, there exist precisely two set pairs $(S_1',H_1'),(S_2',H_2') \in {\mathcal {S}}_1$ with $H_1' = H_2' = H'$ and, in view of ${\mathcal {S}}_1 \prec {\mathcal {S}}_2$, there must exist some $S'' \in {\mathcal {P}}({\mathcal {S}}_2)$ with $S_1' \cup S_2' \cup H' \subseteq S''$. This implies $k \ge 2\ell _1$. Thus, letting $\ell _2$ denote the number of $H'' \in {\mathcal {H}}({\mathcal {S}}_2)$ with $H'' \not \in {\mathcal {H}}({\mathcal {S}}_1)$, we have

$$\begin{aligned} |{\mathcal {P}}({\mathcal {S}}_2)|- |{\mathcal {H}}({\mathcal {S}}_2) |&= |{\mathcal {P}}({\mathcal {S}}_2) |- |{\mathcal {H}}({\mathcal {S}}_1) |+ \ell _1 - \ell _2\\&= |{\mathcal {P}}({\mathcal {S}}_1) |- |{\mathcal {H}}({\mathcal {S}}_1) |+ \ell _1 - \ell _2 - k\\&\le |{\mathcal {P}}({\mathcal {S}}_1) |- |{\mathcal {H}}({\mathcal {S}}_1) |- \ell _1 - \ell _2. \end{aligned}$$

Thus, if $\ell _1 + \ell _2 > 0$, we immediately have $|{\mathcal {P}}({\mathcal {S}}_2) |- |{\mathcal {H}}({\mathcal {S}}_2) |< |{\mathcal {P}}({\mathcal {S}}_1) |- |{\mathcal {H}}({\mathcal {S}}_1) |$. If $\ell _1 + \ell _2 = 0$ we have ${\mathcal {H}}({\mathcal {S}}_1) = {\mathcal {H}}({\mathcal {S}}_2)$. This implies, in view of ${\mathcal {S}}_1 \prec {\mathcal {S}}_2$, that we cannot have ${\mathcal {P}}({\mathcal {S}}_1) = {\mathcal {P}}({\mathcal {S}}_2)$, that is, we must have $k > 0$ and, thus, we also obtain $|{\mathcal {P}}({\mathcal {S}}_2)|- |{\mathcal {H}}({\mathcal {S}}_2)|< |{\mathcal {P}}({\mathcal {S}}_1)|- |{\mathcal {H}}({\mathcal {S}}_1)|$, as required.

Now assume that $(|{\mathcal {P}}({\mathcal {S}}_1)|- |{\mathcal {H}}({\mathcal {S}}_1)|) - (|{\mathcal {P}}({\mathcal {S}}_2)|- |{\mathcal {H}}({\mathcal {S}}_2)|) \ge 2$. First consider the case that there exist two distinct $(S_1'',H_1''),(S_2'',H_2'') \in {\mathcal {S}}_2$ with $|q^{-1}(S_i'',H_i'')|\ge 2$, $i \in \{1,2\}$. Then we put

$$\begin{aligned}{\mathcal {S}}_3 = ({\mathcal {S}}_1 - q^{-1}(S_1'',H_1'')) \cup \{(S_1'',H_1'')\}.\end{aligned}$$

Next consider the case that there exists $(S'',H'') \in {\mathcal {S}}_2$ with $|q^{-1}(S'',H'')|\ge 3$ and $H' = \emptyset$ for all $(S',H') \in q^{-1}(S'',H'')$. Then we select two distinct $(S_1',\emptyset ),(S_2',\emptyset ) \in q^{-1}(S'',H'')$ and put

$$\begin{aligned}{\mathcal {S}}_3 = ({\mathcal {S}}_1 - \{(S_1',\emptyset ),(S_2',\emptyset )\}) \cup \{(S_1' \cup S_2',\emptyset )\}.\end{aligned}$$

The remaining case to consider is that there exists a set pair $(S'',H'') \in {\mathcal {S}}_2$ such that $|q^{-1}(S'',H'')|\ge 4$ and there are three distinct $(S_1',H_1'),(S_2',H_2'),(S_3',H_3') \in q^{-1}(S'',H'')$ with $H_1' = \emptyset$ and $H_2'=H_3'=S_1'$. Then, we put

$$\begin{aligned}{\mathcal {S}}_3 = ({\mathcal {S}}_1 - \{(S_1',H_1'),(S_2',H_2'),(S_3',H_3')\}) \cup \{(S_1' \cup S_2' \cup S_3',\emptyset )\}.\end{aligned}$$

In each case, by construction, we immediately have ${\mathcal {S}}_1 \prec {\mathcal {S}}_3 \prec {\mathcal {S}}_2$. $\square$

## The Poset $({\mathfrak {P}}(X),\preceq )$

In this section, we prove that $\preceq$ is a partial ordering on ${\mathfrak {P}}(X)$. We also give a formula for counting the number of elements in the resulting poset $({\mathfrak {P}}(X),\preceq )$.

We first recall some standard poset concepts (see e.g. [35]). A (finite) *poset* $(M,R)$ consists of a finite non-empty set $M$ and a binary relation $R \subseteq M \times M$ on $M$ that is reflexive, transitive and antisymmetric. An element $m \in M$ is *minimum* (*maximum*) if $(m,a) \in R$ ($(a,m) \in R$) holds for all $a \in M$. A poset is *bounded* if it has a minimum and a maximum element and these elements are then necessarily unique. Two elements $a,b \in M$ are *comparable* if $(a,b) \in R$ or $(b,a) \in R$. A *chain* $C$ is a non-empty subset of $M$ of pairwise comparable elements. The *length* of a chain $C$ is $|C |- 1$. A chain is *maximal* if it is not contained in some strictly longer chain. A poset is *graded* if every maximal chain has the same length. The *height function*^3^$h$ of a graded poset $(M,R)$ assigns to every element $a \in M$ the length $h(a)$ of a longest chain $C$ with $(b,a) \in R$ for all $b \in C$.

### Proposition 1

$({\mathfrak {P}}(X),\preceq )$ is a bounded graded poset with minimum element $\{(\{x\},\emptyset ): x \in X\}$ and maximum element $\{(X,\emptyset )\}$. The height function of this poset is $h: {\mathfrak {P}}(X) \rightarrow \{0,1,\dots ,|X |-1\}$ with $h({\mathcal {S}}) = |X |- |{\mathcal {P}}({\mathcal {S}})|+ |{\mathcal {H}}({\mathcal {S}})|$.

### Proof

We first show that $({\mathfrak {P}}(X),\preceq )$ is a poset. It follows immediately from the definition of the binary relation $\preceq$ that it is reflexive. Moreover, in view of Lemma 6, we cannot have two distinct ${\mathcal {S}}_1,{\mathcal {S}}_2 \in {\mathfrak {P}}(X)$ with ${\mathcal {S}}_1 \preceq {\mathcal {S}}_2$ and ${\mathcal {S}}_2 \preceq {\mathcal {S}}_1$, implying that $\preceq$ is also antisymmetric.

It remains to show that $\preceq$ is transitive. Consider set pair systems ${\mathcal {S}}_1$, ${\mathcal {S}}_2$ and ${\mathcal {S}}_3$ with ${\mathcal {S}}_1 \preceq {\mathcal {S}}_2 \preceq {\mathcal {S}}_3$. Then, in view of (SP1), for all $(S_1,H_1) \in {\mathcal {S}}_1$, there exists some $(S_2,H_2) \in {\mathcal {S}}_2$ with $(S_1,H_1) \le (S_2,H_2)$ and, again in view of (SP1), there also exists some $(S_3,H_3) \in {\mathcal {S}}_3$ with $(S_2,H_2) \le (S_3,H_3)$. By the transitivity of $\le$, we obtain $(S_1,H_1) \le (S_3,H_3)$, as required.

Next consider some $(S_3,H_3) \in {\mathcal {S}}_3$ with $H_3 \ne \emptyset$. First assume that there exists some $(S_2,H_2) \in {\mathcal {S}}_2$ with $H_2=H_3$. Then, by (SP2), there also exists $(S_2,H_2) \in {\mathcal {S}}_2$ with $H_2=H_3$ and $(S_2,H_2) \le (S_3,H_3)$. Now, if there exists some $(S_1,H_1) \in {\mathcal {S}}_1$ with $H_1 = H_2 = H_3$, then, by (SP2), there also exists such a set pair in ${\mathcal {S}}_1$ with $(S_1,H_1) \le (S_2,H_2) \le (S_3,H_3)$. Hence, by the transitivity of $\le$, we have $(S_1,H_1) \le (S_3,H_3)$, as required.

Next assume that there exists no $(S_2,H_2) \in {\mathcal {S}}_2$ with $H_2=H_3$. It suffices to show that this implies that there exists no $(S_1,H_1) \in {\mathcal {S}}_1$ with $H_1=H_3$. So, assume for a contradiction that there exists some $(S_1,H_1) \in {\mathcal {S}}_1$ with $H_1=H_3\ne \emptyset$. Put $H=H_1$. In view of ${\mathcal {S}}_1 \preceq {\mathcal {S}}_2$, there must exist some $(S_2,H_2) \in {\mathcal {S}}_2$ with $(S_1,H) \le (S_2,H_2)$. Note that $H_2 \ne H$ combined with the definition of $\le$ implies $S_1 \cup H \subseteq S_2$ or $S_1 \cup H \subseteq H_2$. Moreover, in view of ${\mathcal {S}}_2 \preceq {\mathcal {S}}_3$, there must exist some $(S_3',H_3') \in {\mathcal {S}}_3$ with $(S_2,H_2) \le (S_3',H_3')$. This implies that $S_1 \cup H \subseteq S_3'$ or $S_1 \cup H \subseteq H_3'$. But then, $H \subsetneq S_3'$ or $H \subsetneq H_3'$ must hold in contradiction to (PL1). Thus, ${\mathcal {S}}_1 \preceq {\mathcal {S}}_3$ holds, establishing that $\preceq$ is transitive and, thus, $({\mathfrak {P}}(X),\preceq )$ is a poset.

Next, we show that $\{(\{x\},\emptyset ): x \in X\}$ and $\{(X,\emptyset )\}$ are the minimum and maximum element, respectively, in $({\mathfrak {P}}(X),\preceq )$. Clearly, $\{(\{x\},\emptyset ): x \in X\}$ and $\{(X,\emptyset )\}$ are both polestar systems and, thus, elements of ${\mathfrak {P}}(X)$. Consider any ${\mathcal {S}} \in {\mathfrak {P}}(X)$. Then, for all $(S,H) \in {\mathcal {S}}$, we have $S \cup H \subseteq X$, implying $(S,H) \le (X,\emptyset )$ and, thus, ${\mathcal {S}} \preceq \{(X,\emptyset )\}$. Similarly, in view of (PL1), for all $x \in X$, there must exist some $(S,H) \in {\mathcal {S}}$ with $x \in S$, implying that $(\{x\},\emptyset ) \le (S,H)$. Thus, $\{(\{x\},\emptyset ): x \in X\} \preceq {\mathcal {S}}$. It follows that $({\mathfrak {P}}(X),\preceq )$ is a bounded poset.

That $({\mathfrak {P}}(X),\preceq )$ is a graded poset with height function $h$ is now an immediate consequence of Lemma 6 in view of $h(\{(\{x\},\emptyset ): x \in X\}) = 0$ and $h(\{(X,\emptyset )\}) = |X |-1$. $\square$

The next corollary describes the relationship between $({\mathfrak {P}}(X),\preceq )$ and the poset $({\mathfrak {B}}(X),\sqsubseteq )$ of partitions of $X$. Two posets $(M_1,R_1)$ and $(M_2,R_2)$ are *isomorphic* if there exists a bijective map $f: M_1 \rightarrow M_2$ such that, for all $a,b \in M_1$, $(a,b) \in R_1$ if and only if $(f(a),f(b)) \in R_2$.

### Corollary 1

The restriction of the poset $({\mathfrak {P}}(X),\preceq )$ to those ${\mathcal {S}} \in {\mathfrak {P}}(X)$ with ${\mathcal {H}}({\mathcal {S}}) = \emptyset$ is isomorphic to the poset $({\mathfrak {B}}(X),\sqsubseteq )$ of partitions of $X$.

### Proof

We map any ${\mathcal {S}} \in {\mathfrak {P}}(X)$ with ${\mathcal {H}}({\mathcal {S}}) = \emptyset$ to the partition ${\mathcal {P}}({\mathcal {S}}) \in {\mathfrak {B}}(X)$. This map is bijective. Moreover, for ${\mathcal {S}}_1,{\mathcal {S}}_2 \in {\mathfrak {P}}(X)$ with ${\mathcal {H}}({\mathcal {S}}_1) = {\mathcal {H}}({\mathcal {S}}_2) = \emptyset$ we have ${\mathcal {S}}_1 \preceq {\mathcal {S}}_2$ if and only if for all $A_1 \in {\mathcal {P}}({\mathcal {S}}_1)$ there exists some $A_2 \in {\mathcal {P}}({\mathcal {S}}_2)$ with $A_1 \subseteq A_2$, as required. $\square$

In the remaining part of this section, we give a formula for the number $\lambda _n = |{\mathfrak {P}}(X)|$ of polestar systems on a set $X$ with $n \ge 1$ elements. The values of $\lambda _n$ for $n = 1,2,\dots ,8$ are 1, 2, 8, 45, 277, 1853, 14065, 122118. For $k \in \{1,2,\dots ,n\}$, we denote by $\alpha _{n,k}$ the *Stirling number* of the second kind, that is, the number of partitions of $X$ into $k$ subsets. In addition, for $\ell \in \{0,1,\dots ,\lfloor \frac{k}{3} \rfloor \}$, we denote by $\beta _{k,\ell }$ the number of partitions of a set with $k$ elements into $\ell$ subsets with three elements and $k-3\ell$ subsets with one element. It is known [31] that

$$\begin{aligned}\beta _{k,\ell } = \frac{k!}{6^{\ell } \cdot \ell ! \cdot (k-3\ell )!}.\end{aligned}$$

### Proposition 2

For all $n \ge 1$ we have

1 $$\begin{aligned} \lambda _n = \sum _{k=1}^n \alpha _{n,k} \cdot \left( \sum _{\ell =0}^{\lfloor \frac{k}{3} \rfloor } \beta _{k,\ell } \cdot 3^{\ell } \right) . \end{aligned}$$

### Proof

Let $X$ be a set with $n \ge 1$ elements. Consider ${\mathcal {S}} \in {\mathfrak {P}}(X)$ and put $k =|{\mathcal {P}}({\mathcal {S}})|$. By the definition of a polestar system, ${\mathcal {S}}$ arises from ${\mathcal {P}}({\mathcal {S}})$ by forming, for some $\ell \in \{0,1,\dots ,\lfloor \frac{k}{3} \rfloor \}$, a partition $\Pi ({\mathcal {P}}({\mathcal {S}}))$ of ${\mathcal {P}}({\mathcal {S}})$ into $\ell$ subsets with three elements and $k-3\ell$ subsets with one element. Each 1-element set $\{S\} \in \Pi ({\mathcal {P}}({\mathcal {S}}))$ yields the set pair $(S,\emptyset )$. For each 3-element set $\{S_1,S_2,S_3\} \in \Pi ({\mathcal {P}}({\mathcal {S}}))$ we select $i \in \{1,2,3\}$ and obtain the three set pairs $(S_i,\emptyset )$, $(S_j,S_i)$, $j \in \{1,2,3\} - \{i\}$.

Formula (1) directly reflects the process described above for obtaining a polestar system from a fixed partition of $X$ into $k$ subsets. In view of the fact that every partition of $X$ yields a different collection of polestar systems on $X$, we form the outer sum over the values of $k$. The inner sum then accounts for the number of polestar systems that arise from any fixed partition of $X$ into $k$ subsets. $\square$

## Encoding Ranked *X*-Cactuses

In this section, we show in Theorem 2 that we can encode (isomorphism classes) of ranked $X$-cactuses in terms of the chains in the poset $({\mathfrak {P}}(X),\preceq )$. We begin by giving a precise statement of this result. We call two equidistant $X$-cactuses $({\mathcal {N}}'=((V',A'),\varphi '),t')$ and $({\mathcal {N}}''=((V'',A''),\varphi ''),t'')$ *isomorphic* if there exists a DAG-isomorphism $f:V' \rightarrow V''$ such that

(IC1): $f(\varphi '(x)) = \varphi ''(x)$ for all $x \in X$ and
(IC2): $t'(v) = t''(f(v))$ for all $v \in V'$.

Note that this definition includes isomorphisms between ranked $X$-cactuses as a special case. For rooted $X$-cactuses without a time-stamp function to be isomorphic, condition (IC2) is not required. We now state the aforementioned result.

### Theorem 2

There is a one-to-one correspondence between chains in the poset $({\mathfrak {P}}(X),\preceq )$ that contain the maximum element $\{(X,\emptyset )\}$ and (isomorphism classes of) ranked $X$-cactuses. The length of the chain equals the size of the ranking of the corresponding ranked $X$-cactus. Maximal chains correspond to binary ranked $X$-cactuses with rankings of size $|X |-1$.

To prove this theorem, note that by Lemmas 3 and 4, every ranked $X$-cactus corresponds to a chain ${\mathfrak {C}}$ in $({\mathfrak {P}}(X),\preceq )$ with $\{(X,\emptyset )\} \in {\mathfrak {C}}$. Moreover, by Lemma 2, we have $|{\mathfrak {C}}|\le |X |- 1$ for such a chain with equality holding if and only if the ranked $X$-cactus is binary. Thus, to prove Theorem 2, it suffices to show that for all chains ${\mathfrak {C}}$ in $({\mathfrak {P}}(X),\preceq )$ with $\{(X,\emptyset )\} \in {\mathfrak {C}}$ there exists, up to isomorphism, a unique ranked $X$-cactus $({\mathcal {N}},r)$ with ${\mathfrak {C}} = \{{\mathcal {S}}_i({\mathcal {N}}): 0 \le i \le \sigma (r)\}$. This follows immediately from Lemmas 7 and 8 below, and will be done in two steps. First, for any chain ${\mathfrak {C}} \subseteq {\mathfrak {P}}(X)$ with $\{(X,\emptyset )\} \in {\mathfrak {C}}$, we form the set pair system ${\mathcal {S}}({\mathfrak {C}}) = \bigcup _{{\mathcal {S}}' \in {\mathfrak {C}}} {\mathcal {S}}'$ consisting of all set pairs that occur in the polestar systems in ${\mathfrak {C}}$ and construct a suitable rooted, compressed, phylogenetic $X$-cactus ${\mathcal {N}}({\mathfrak {C}})$ (see Lemma 7). Second, we perform some technical modifications on ${\mathcal {N}}({\mathfrak {C}})$, if necessary, to obtain ${\mathcal {N}}$ and then construct a suitable ranking $r$ (see Lemma 8).

### Lemma 7

For all chains ${\mathfrak {C}}$ in $({\mathfrak {P}}(X),\preceq )$ with $\{(X,\emptyset )\} \in {\mathfrak {C}}$ there exists, up to isomorphism, a unique rooted, compressed, phylogenetic $X$-cactus ${\mathcal {N}}({\mathfrak {C}})$ with ${\mathcal {S}}({\mathcal {N}}({\mathfrak {C}}))$ $= {\mathcal {S}}({\mathfrak {C}}) \cup \{(\{x\},\emptyset ): x \in X\}$.

### Proof

Put ${\mathcal {S}} = {\mathcal {S}}({\mathfrak {C}}) \cup \{(\{x\},\emptyset ): x \in X\}$. We show below that ${\mathcal {S}}$ satisfies certain properties (NC1)–(NC5). We do this to then apply [22, Theorem 5], which states that if a set pair system ${\mathcal {S}}'$ on $X$ has these properties there exists, up to isomorphism, a unique rooted, compressed, phylogenetic $X$-cactus ${\mathcal {N}}({\mathcal {S}}')$ with ${\mathcal {S}}' = {\mathcal {S}}({\mathcal {N}}({\mathcal {S}}'))$, as required. In the following, we first state each of the properties (NC1)–(NC5) and then verify that ${\mathcal {S}}$ has this property.

(NC1)—$$(X,\emptyset ) \in {\mathcal {S}}$$:

This is clearly the case.

(NC2)—$(\{x\},\emptyset ) \in {\mathcal {S}}$, for all $x \in X$:

By construction of ${\mathcal {S}}$, this is the case.

(NC3)—For every $(S,H) \in {\mathcal {S}}$ with $H \ne \emptyset$, we have $(H,\emptyset ) \in {\mathcal {S}}$:

Consider any $(S,H) \in {\mathcal {S}}$ with $H \ne \emptyset$. Then, by construction, there must exist some ${\mathcal {S}}' \in {\mathfrak {C}}$ with $(S,H) \in {\mathcal {S}}'$. In view of (PL3) we must have $(H,\emptyset ) \in {\mathcal {S}}'$. Thus, by the definition of ${\mathcal {S}}$, it follows that $(H,\emptyset ) \in {\mathcal {S}}$, as required.

(NC4)—For any two distinct $(S_1,H_1), (S_2,H_2) \in {\mathcal {S}}$ one of (i) $(S_1,H_1) < (S_2,H_2)$, (ii) $(S_2,H_2) < (S_1,H_1)$, (iii) $(S_1 \cup H_1) \cap (S_2 \cup H_2) = \emptyset$, or (iv) $S_1 \cap S_2 = \emptyset$ and $H_1 = H_2 \ne \emptyset$ holds:

Consider $(S_1,H_1), (S_2,H_2) \in {\mathcal {S}}$ with $(S_1,H_1) \ne (S_2,H_2)$. By construction, there must exist ${\mathcal {S}}_1,{\mathcal {S}}_2 \in {\mathfrak {C}}$ with $(S_1,H_1) \in {\mathcal {S}}_1$ and $(S_2,H_2) \in {\mathcal {S}}_2$. Without loss of generality, we may assume that ${\mathcal {S}}_1 \preceq {\mathcal {S}}_2$.

First we consider the case ${\mathcal {S}}_1 = {\mathcal {S}}_2$. Then, in view of (PL1) and (PL2), we have $S_1 \cap S_2 = \emptyset$. Thus, if $H_1 = H_2 \ne \emptyset$, we are done. Otherwise, in view of (PL3) and (PL1), we must have $H_1 \cap H_2 = \emptyset$ and, thus, $(S_1 \cup H_1) \cap (S_2 \cup H_2) = \emptyset$, as required.

Next consider the case ${\mathcal {S}}_1 \prec {\mathcal {S}}_2$. Then there must exist some $(S,H) \in {\mathcal {S}}_2$ with $(S_1,H_1) \le (S,H)$. If $(S,H) = (S_2,H_2)$ we immediately have $(S_1,H_1) \le (S_2,H_2)$ and are done. Therefore, assume $(S,H) \ne (S_2,H_2)$. In view (PL2), this implies $S \cap S_2 = \emptyset$. Thus, by the definition of $\le$ one of the following must hold:

- $S_1 \cup H_1 \subseteq S$: Then, by the definition of set pairs, $(S_1 \cup H_1) \cap H = \emptyset$ and, in view of $S \cap S_2 = \emptyset$, also $(S_1 \cup H_1) \cap S_2 = \emptyset$. Thus, if $S \cap H_2 = \emptyset$ we have $(S_1 \cup H_1) \cap (S_2 \cup H_2) = \emptyset$. Therefore, assume that $S=H_2$. Then, we have $S_1 \cup H_1 \subseteq H_2$ implying $(S_1,H_1) < (S_2,H_2)$.
- $S_1 \cup H_1 \subseteq H$: Then, if $H=H_2$ or $H=S_2$, we immediately have $(S_1,H_1) < (S_2,H_2)$. Otherwise, we must have $H \cap H_2 = \emptyset$ and $H \cap S_2 = \emptyset$ and, thus, $(S_1 \cup H_1) \cap (S_2 \cup H_2) = \emptyset$.
- $S_1 \subsetneq S$ and $H_1 = H \ne \emptyset$: First note that this implies $S \cap H_2 = \emptyset$ because otherwise we would have $(S,\emptyset ) \in {\mathcal {S}}_2$ in view of (PL3), which is impossible in view of $(S,H) \in {\mathcal {S}}_2$ and (PL2). Also note that if $H=S_2$ we must have $(S_2,H_2) = (H,\emptyset )$ in view of (PL2), implying that $(S_1,H_1) < (S_2,H_2)$. Finally, if $H \cap S_2 = \emptyset$, we obtain $(S_1 \cup H_1) \cap (S_2 \cup H_2) = \emptyset$.

This establishes that ${\mathcal {S}}$ satisfies (NC4).

(NC5)—There are no three distinct $(S_1,H_1)$, $(S_2,H_2)$, $(S_3,H_3) \in {\mathcal {S}}$ with $H_1 = H_2 = H_3 \ne \emptyset$, $S_1 \cap S_2 = \emptyset$ and either $S_1 \cup S_2 \subseteq S_3$ or $(S_1 \cup S_2) \cap S_3 = \emptyset$:

Consider ${\mathcal {S}}_1, {\mathcal {S}}_2, {\mathcal {S}}_3 \in {\mathfrak {C}}$ with ${\mathcal {S}}_1 \preceq {\mathcal {S}}_2 \preceq {\mathcal {S}}_3$. Assume that there exist set pairs $(S_1',H),(S_1'',H) \in {\mathcal {S}}_1$, $(S_2',H),(S_2'',H) \in {\mathcal {S}}_2$ and $(S_3',H),(S_3'',H) \in {\mathcal {S}}_3$ with $H \ne \emptyset$. By (PL3), there are precisely these two set pairs contained in each of ${\mathcal {S}}_1$, ${\mathcal {S}}_2$ and ${\mathcal {S}}_3$ for the fixed set $H$. In view of (SP2), we may assume without loss of generality that $(S_1',H) \le (S_2',H) \le (S_3',H)$ and $(S_1'',H) \le (S_2'',H) \le (S_3'',H)$, implying that we have $S_1' \subseteq S_2' \subseteq S_3'$ and $S_1'' \subseteq S_2'' \subseteq S_3''$. But then, it is impossible to select three distinct set pairs $(S_1,H), (S_2,H), (S_3,H)$ from among

$$\begin{aligned}(S_1',H),(S_1'',H),(S_2',H),(S_2'',H),(S_3',H),(S_3'',H)\end{aligned}$$

with $S_1 \cap S_2 = \emptyset$ and either $S_1 \cup S_2 \subseteq S_3$ or $(S_1 \cup S_2) \cap S_3 = \emptyset$. This establishes that ${\mathcal {S}}$ satisfies (NC5). $\square$

Recall from Sect. 2 that, for every rooted $X$-cactus ${\mathcal {N}}$, we denote by $\widehat{{\mathcal {N}}}^*$ the associated rooted, compressed, phylogenetic $X$-cactus.

### Lemma 8

For all chains ${\mathfrak {C}}$ in $({\mathfrak {P}}(X),\preceq )$ with $\{(X,\emptyset )\} \in {\mathfrak {C}}$, there exists, up to isomorphism, a unique ranked $X$-cactus $({\mathcal {N}},r)$ such that $\widehat{{\mathcal {N}}}^* = {\mathcal {N}}({\mathfrak {C}})$ and ${\mathfrak {C}} = \{{\mathcal {S}}_i({\mathcal {N}}): 0 \le i \le \sigma (r)\}$.

### Proof

Let ${\mathcal {N}}({\mathfrak {C}}) = (({\widehat{V}}^*,{\widehat{A}}^*),{\widehat{\varphi }}^*)$ be the rooted, compressed, phylogenetic $X$-cactus that exists by Lemma 7. To obtain a rooted $X$-cactus ${\mathcal {N}} = ((V,A),\varphi )$ with ${\mathcal {S}}({\mathcal {N}}) = {\mathcal {S}}({\mathfrak {C}})$, we take ${\mathcal {N}}({\mathfrak {C}})$ and modify it. The first modification applies to all $x \in X$ with $(\{x\},\emptyset ) \not \in {\mathcal {S}}({\mathfrak {C}})$ and corresponds to reversing the addition of leaves that was illustrated in Fig. 2b. For each such $x$, we contract the arc $(u,v) \in {\widehat{A}}^*$ with $v = {\widehat{\varphi }}^*(x)$ and put $\varphi (x) = u$. The second modification applies to all set pairs $(S,\emptyset ) \in {\mathcal {S}}({\mathfrak {C}})$ such that $(S,\emptyset ) \in {\mathcal {S}}_1 \cap {\mathcal {S}}_2$ for ${\mathcal {S}}_1, {\mathcal {S}}_2 \in {\mathfrak {C}}$ with ${\mathcal {S}}_1 \prec {\mathcal {S}}_2$, $S \not \in {\mathcal {H}}({\mathcal {S}}_1)$ and $S \in {\mathcal {H}}({\mathcal {S}}_2)$. This implies, in view of the definition of the polestar systems ${\mathcal {S}}_i({\mathcal {N}})$, $0 \le i \le |X |-1$, that we need to modify ${\mathcal {N}}({\mathfrak {C}})$ to ensure that ${\mathcal {N}}$ contains two distinct vertices $u$ and $v$ with $(S(u),H(u)) = (S(v),H(v)) = (S,\emptyset )$. This corresponds to reversing the compression that was illustrated in Fig. 2c. Thus, in view of (SH2), for each such set pair $(S,H)$, we locate the vertex $u \in {\widehat{V}}^*$ with $(S(u),H(u)) = (S,\emptyset )$ and then expand the vertex $u$ into an arc $(u,v)$ such that the outgoing arcs of $u$ become the outgoing arcs of $v$ and, for all $x \in X$ with $u={\widehat{\varphi }}^*(x)$, we put $\varphi (x) = v$.

Note that the resulting rooted $X$-cactus ${\mathcal {N}}$ need no longer be phylogenetic or compressed and that for all ranked $X$-cactuses $({\mathcal {N}}',r')$ with ${\mathfrak {C}} = \{{\mathcal {S}}_i({\mathcal {N}}'): 0 \le i \le \sigma (r)'\}$ we necessarily have that ${\mathcal {N}}'$ is isomorphic to ${\mathcal {N}}$ in view of the fact that $\widehat{{\mathcal {N}}'}^*$ and $\widehat{{\mathcal {N}}}^*$ must be isomorphic by Lemma 7.

Thus, it remains to show that there exists a unique ranking $r$ of the vertices of ${\mathcal {N}}$ to obtain a ranked $X$-cactus $({\mathcal {N}},r)$ with ${\mathfrak {C}} = \{{\mathcal {S}}_i({\mathcal {N}}): 0 \le i \le \sigma (r)\}$. Let $c$ denote the length of ${\mathfrak {C}}$ and consider the sequence ${\mathcal {S}}_0 \prec {\mathcal {S}}_1 \prec \dots \prec {\mathcal {S}}_c$ of the polestar systems in ${\mathfrak {C}}$. The value $r(u)$ for a vertex $u \in V$ that is not a reticulation vertex is defined by considering the set pair $(S(u),H(u))$ and putting $r(u)$ to be the smallest index $0 \le i \le c$ with $(S(u),H(u)) \in {\mathcal {S}}_i$. Note that this is the only available choice for the rank of $u$. The value $r(u)$ of a reticulation vertex $u$ is defined to be equal to the rank of the parents of $u$, which, since ${\mathcal {N}}$ is a rooted $X$-cactus, cannot be reticulation vertices and have been assigned a rank already.

Next, we show that the map $r: V \rightarrow \{0,1,\dots ,c\}$ defined above is a ranking of the vertices of ${\mathcal {N}}$. First note that the value $r(u)$ of a reticulation vertex $u$ is well-defined. Indeed, in view of (SH3), we must have $r(p_1) = r(p_2)$ for the two parents $p_1$ and $p_2$ of $u$, that is, the set pairs $(S(p_1),H(p_1))$ and $(S(p_2),H(p_2))$ with $H(p_1) = H(p_2) = H$ are both contained in the polestar system ${\mathcal {S}}_i$ with the smallest index $i$ such that $H \in {\mathcal {H}}({\mathcal {S}}_i)$. This establishes (TS3).

To establish (TS1), consider any $x \in X$. By (PL1) there exists a unique set pair $(S,H) \in {\mathcal {S}}_0$ with $x \in S$. Then, by Lemma 5, it suffices to consider the following two cases:

- There is precisely one $(S',H') \in {\mathcal {S}}({\mathfrak {C}})$ with $(S',H') < (S,H)$. Then, we must have $(S',H') \in {\mathcal {S}}_0$, $H' = \emptyset$ and $H=S'$. This implies that there exists a reticulation vertex $u$ in ${\mathcal {N}}$ that is a leaf with $(S(u),H(u)) = (S',H')$ and that $u$ is the single child of a vertex $p$ with $(S(p),H(p)) = (S,H)$. Since $x \in S$ and $S \cap S' = \emptyset$, we have $\varphi (x) = p$. By construction, we have $r(p) = 0$, as required.
- There is no $(S',H') \in {\mathcal {S}}({\mathfrak {C}})$ with $(S',H') < (S,H)$. Then, there exists a leaf $u$ of ${\mathcal {N}}$ with $(S(u),H(u)) = (S,H)$ and we must have $\varphi (x) = u$. Again, by construction, we have $r(u) = 0$, as required.

Now, we turn to (TS2). Consider an arc $(u,v)$ of ${\mathcal {N}}$ such that $v$ is not a reticulation vertex. As mentioned in Sect. 5.1, since $v$ is a descendant of $u$, we have $(S(v),H(v)) \le (S(u),H(u))$. If $(S(v),H(v)) = (S(u),H(u))$ then, by the construction of ${\mathcal {N}}$ from ${\mathcal {N}}({\mathfrak {C}})$, $u$ is a reticulation vertex whose single child is $v$ and there exist $0 \le i < j \le c$ with $r(v) = i$ and $r(u) = r(p_1) = r(p_2) = j$, where $p_1$ and $p_2$ are the two parents of $u$. Similarly, in view of Lemma 5, if $(S(v),H(v)) < (S(u),H(u))$ there also exist $0 \le i < j \le c$ with $r(v) = i$ and $r(u) = j$. This establishes (TS2).

The last property required for the map $r$ to be a ranking is that, for all $j \in \{0,1,\dots ,c\}$, there exists a vertex $u$ of ${\mathcal {N}}$ with $r(u) = j$. (TS1) implies that this is the case for $j=0$. Therefore, consider $j \ge 1$. Then, in view of Lemma 6, there exists some $(S,H) \in {\mathcal {S}}_j$ with $(S,H) \not \in {\mathcal {S}}_i$ for all $i < j$. Let $u$ be a vertex of ${\mathcal {N}}$ with $(S(u),H(u)) = (S,H)$. If $u$ is not a reticulation vertex, we have $r(u) = j$. If $u$ is a reticulation vertex, we have $r(u) = r(p_1) = r(p_2) = j$ for the two parents $p_1$ and $p_2$ of $u$ since, by (PL3), $(S(p_1),H(p_1))$ and $(S(p_2),H(p_2))$ are also both contained in ${\mathcal {S}}_j$ but not in ${\mathcal {S}}_i$ for all $i < j$.

To finish the proof of the lemma, we show that ${\mathcal {S}}_j = {\mathcal {S}}_j({\mathcal {N}})$ for all $j \in \{0,1,\dots ,c\}$. We clearly have ${\mathcal {S}}_c = \{(X,\emptyset )\} = {\mathcal {S}}_c({\mathcal {N}})$. Consider $j < c$. In view of Lemma 4, (PL1) and (PL2) it suffices to show that, for all $u \in V_j$, we have $(S(u),H(u)) \in {\mathcal {S}}_j$. Let $p$ be the unique parent of $u$. By the definition of $V_j$ given in Sect. 5.1, we have $r(u) = i \le j$ and $r(p) = k > j$. In particular, we have $(S(u),H(u)) \in {\mathcal {S}}_i$ and $(S(p),H(p)) \in {\mathcal {S}}_k$. In view of ${\mathcal {S}}_i \preceq {\mathcal {S}}_j \prec {\mathcal {S}}_k$ there must exist $(S',H') \in {\mathcal {S}}_j$ with $(S(u),H(u)) \le (S',H')$ and also some $(S'',H'') \in {\mathcal {S}}_k$ with $(S',H') \le (S'',H'')$. Since $p$ is the parent of $u$ we have $(S(u),H(u)) \le (S(p),H(p))$ and, since all set pairs in ${\mathcal {S}}({\mathfrak {C}})$ correspond to at least one vertex of ${\mathcal {N}}$, we must necessarily have $(S'',H'') = (S(p),H(p))$. It follows that either $(S(u),H(u)) = (S',H') = (S(p),H(p))$ or $(S(u),H(u)) = (S',H') < (S(p),H(p))$ holds, implying $(S(u),H(u)) \in {\mathcal {S}}_j$, as required. $\square$

As an immediate consequence of Theorem 2 we obtain Theorem 1, which we restate in the following corollary using poset terminology.

### Corollary 2

There is a one-to-one correspondence between chains in the graded poset $({\mathfrak {B}}(X),\sqsubseteq )$ that contain $\{X\}$ and isomorphism classes of ranked $X$-trees.

### Proof

In view of the fact that a rooted $X$-cactus ${\mathcal {N}}$ is a rooted $X$-tree if and only if the associated set pair system ${\mathcal {S}}({\mathcal {N}})$ does not contain a set pair $(S,H)$ with $H \ne \emptyset$, it follows by Theorem 2 that ranked $X$-trees correspond to chains ${\mathfrak {C}}$ in the poset $({\mathfrak {P}}(X),\preceq )$ with $\{(X,\emptyset )\} \in {\mathfrak {C}}$ and ${\mathcal {H}}({\mathcal {S}})=\emptyset$ for all ${\mathcal {S}} \in {\mathfrak {C}}$. This implies, by Corollary 1, that ranked $X$-trees correspond to chains in the poset $({\mathfrak {B}}(X),\sqsubseteq )$ that contain the partition $\{X\}$. $\square$

## The Space of Equidistant *X*-Cactuses

We now define equidistant-cactus space, ${\mathfrak {N}}(X)$, and show that it is a CAT(0)-metric space. The construction of ${\mathfrak {N}}(X)$ follows the outline presented at the start of Sect. 5. More specifically, we put ${\mathfrak {P}}^{\circ }(X) = {\mathfrak {P}}(X) - \{\{(X,\emptyset )\}\}$ and let ${\mathcal {F}}(\preceq )$ denote the set of chains in the subposet $({\mathfrak {P}}^{\circ }(X),\preceq )$ of the poset $({\mathfrak {P}}(X),\preceq )$. We then define ${\mathfrak {N}}(X)$ to be the orthant space of the order complex of $({\mathfrak {P}}^{\circ }(X),\preceq )$. Figure 7 gives an example of the structure of ${\mathfrak {N}}(X)$ for $X=\{a,b,c\}$.

### Theorem 3

The orthant space ${\mathfrak {N}}(X) = ({\mathfrak {M}}_{({\mathfrak {P}}^{\circ }(X),{\mathcal {F}}(\preceq ))},D_{({\mathfrak {P}}^{\circ }(X),{\mathcal {F}}(\preceq ))})$ is a $\text {CAT}(0)$-metric space whose points are in one-to-one correspondence with isomorphism classes of equidistant $X$-cactuses.

### Proof

As an immediate consequence of the definition of a chain as a set of pairwise comparable elements in a poset, we have that $({\mathfrak {P}}^{\circ }(X),{\mathcal {F}}(\preceq ))$ is a flag complex (cf. Section 4.1). Hence, $({\mathfrak {M}}_{({\mathfrak {P}}^{\circ }(X),{\mathcal {F}}(\preceq ))},D_{({\mathfrak {P}}^{\circ }(X),{\mathcal {F}}(\preceq ))})$ is a $\text {CAT}(0)$-metric space.

It remains to show that the points of ${\mathfrak {M}}_{({\mathfrak {P}}^{\circ }(X),{\mathcal {F}}(\preceq ))}$ are in one-to-one correspondence with isomorphism classes of equidistant $X$-cactuses. Every $\omega \in {\mathfrak {M}}_{({\mathfrak {P}}^{\circ }(X),{\mathcal {F}}(\preceq ))}$ corresponds, up to isomorphism, to a unique equidistant $X$-cactus $({\mathcal {N}},t)$ as follows. Put $\sigma = |\text {supp}(\omega ) |$ and ${\mathfrak {C}} = \text {supp}(\omega ) \cup \{\{(X,\emptyset )\}\}$. Note that ${\mathfrak {C}}$ is a chain in the poset $({\mathfrak {P}}(X),\preceq )$. Consider the sequence

$$\begin{aligned}{\mathcal {S}}_0 \prec {\mathcal {S}}_1 \prec {\mathcal {S}}_2 \prec \dots \prec {\mathcal {S}}_{\sigma } = \{(X,\emptyset )\}\end{aligned}$$

of the set pair systems in ${\mathfrak {C}}$. By Theorem 2, there exists, up to isomorphism, a unique ranked $X$-cactus $({\mathcal {N}}=((V,A),\varphi ),r)$ with $\sigma (r) = \sigma$ and ${\mathcal {S}}_i = {\mathcal {S}}_i({\mathcal {N}})$ for all $i \in \{0,1,2,\dots ,\sigma \}$. The time-stamp function $t$ on the vertices of ${\mathcal {N}}$ is then defined by putting

$$\begin{aligned}t(v) = {\left\{ \begin{array}{ll} 0 &{}\quad \text {if} \ r(v) = 0\\ \sum _{i=0}^{r(v)-1} \omega ({\mathcal {S}}_i) &{}\quad \text {if} \ r(v) > 0 \end{array}\right. }\end{aligned}$$

for all $v \in V$. Note that every $\omega ' \in {\mathfrak {M}}_{({\mathfrak {P}}^{\circ }(X),{\mathcal {F}}(\preceq ))}$ with $\omega ' \ne \omega$ and $\text {supp}(\omega ') = \text {supp}(\omega )$ yields the same ranked $X$-cactus $({\mathcal {N}},r)$ but a time-stamp function $t' \ne t$ on the vertices of ${\mathcal {N}}$. Also note that every equidistant $X$-cactus $({\mathcal {N}},t)$ arises from some $\omega \in {\mathfrak {M}}_{({\mathfrak {P}}^{\circ }(X),{\mathcal {F}}(\preceq ))}$ as described above. $\square$

To illustrate the proof of Theorem 3, consider the equidistant $X$-cactus $({\mathcal {N}},t)$ on $X=\{a,b,c,d,e\}$ in Fig. 3, which arises from the point $\omega \in {\mathfrak {M}}_{({\mathfrak {P}}^{\circ }(X),{\mathcal {F}}(\preceq ))}$ with $\text {supp}(\omega ) = \{{\mathcal {S}}_0,{\mathcal {S}}_1,{\mathcal {S}}_2,{\mathcal {S}}_3\}$, where

$$\begin{aligned} {\mathcal {S}}_3&= \{(\{a\},\{b\}),(\{b\},\emptyset ),(\{c,d,e\},\{b\})\}\\ {\mathcal {S}}_2&= \{(\{a\},\{b\}),(\{b\},\emptyset ),(\{c\},\{b\}),(\{d,e\},\emptyset )\}\\ {\mathcal {S}}_1&= \{(\{a\},\{b\}),(\{b\},\emptyset ),(\{c\},\{b\}),(\{d\},\emptyset ),(\{e\},\emptyset )\}\\ {\mathcal {S}}_0&= \{(\{a\},\emptyset ),(\{b\},\emptyset ),(\{c\},\emptyset ),(\{d\},\emptyset ),(\{e\},\emptyset )\}, \end{aligned}$$

and $$\omega ({\mathcal {S}}_0) = 0.8$$, $$\omega ({\mathcal {S}}_1) = 0.4$$, $$\omega ({\mathcal {S}}_2) = 1.2$$, $$\omega ({\mathcal {S}}_3) = 0.6$$.

**Fig. 8:**
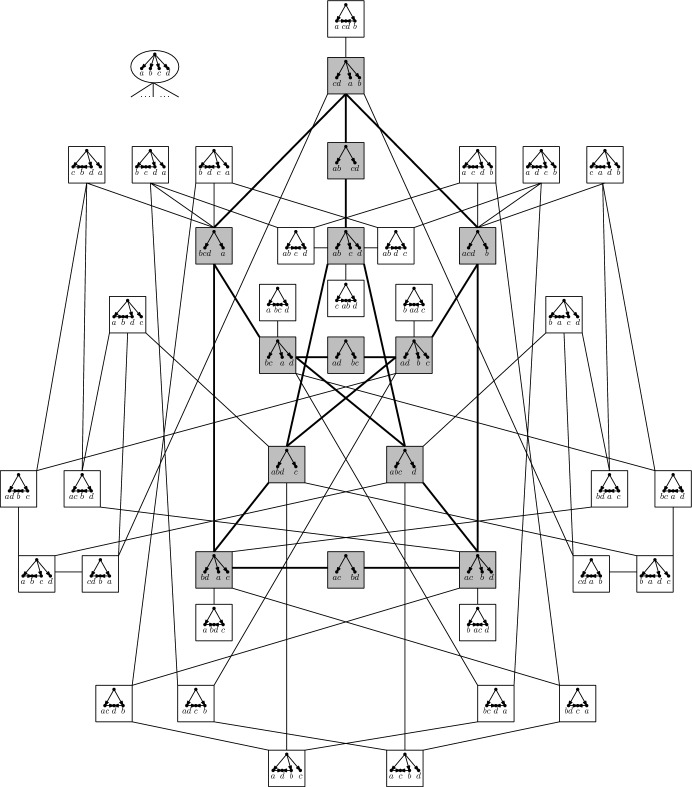
The graph that determines the structure of the link of the origin ${\mathfrak {L}}_{({\mathfrak {P}}^{\circ }(X),{\mathcal {F}}(\preceq ))}$ for $X=\{a,b,c,d\}$. The oval vertex is adjacent to all other vertices. The ranked $X$-cactus displayed for each vertex corresponds to the chain $\{{\mathcal {S}},\{(X,\emptyset )\}\}$ for each ${\mathcal {S}} \in {\mathfrak {P}}^{\circ }(X)$

In general, as equidistant-cactus space is high-dimensional, for $|X |\ge 4$ its structure is not easy to visualize. However, to get some insights it can be useful to consider the so-called *link of the origin*

$$\begin{aligned}{\mathfrak {L}}_{({\mathfrak {P}}^{\circ }(X),{\mathcal {F}}(\preceq ))} = \left\{ \omega \in {\mathfrak {M}}_{({\mathfrak {P}}^{\circ }(X),{\mathcal {F}}(\preceq ))}: \sum _{{\mathcal {S}} \in {\mathfrak {P}}^{\circ }(X)} \omega ({\mathcal {S}}) = 1\right\} ,\end{aligned}$$

a geometric realization of the abstract simplicial complex $({\mathfrak {P}}^{\circ }(X),{\mathcal {F}}(\preceq ))$. Since $({\mathfrak {P}}^{\circ }(X),{\mathcal {F}}(\preceq ))$ is a flag complex, the structure of ${\mathfrak {L}}_{({\mathfrak {P}}^{\circ }(X),{\mathcal {F}}(\preceq ))}$ is completely determined by the graph with vertex set ${\mathfrak {P}}^{\circ }(X)$ in which two distinct vertices are connected by an edge if and only if they are comparable by $\preceq$. In Fig. 8, we present the link of the origin of ${\mathfrak {N}}(X)$ for $|X |=4$. Note that, for this case, we have $|{\mathfrak {P}}^{\circ }(X)|= 44$ and that there are 14 vertices that correspond to rooted $X$-trees. The shaded vertices in Fig. 8 together with the oval vertex induce a subgraph that is isomorphic to the graph corresponding to the link of the origin of $\tau$-space (i.e. ${\mathfrak {M}}_{({\mathfrak {B}}^{\circ }(X),{\mathcal {F}}(\sqsubseteq ))}$), which is isomorphic to a subdivision of the Petersen graph (see also [18, Fig. 3]).

We conclude this section with a corollary of Theorem 3 that describes a relationship between $\tau$-space and equidistant-cactus space.

### Corollary 3

The orthants of ${\mathfrak {M}}_{({\mathfrak {B}}^{\circ }(X),{\mathcal {F}}(\sqsubseteq ))}$ are in one-to-one correspondence with the orthants ${\mathfrak {O}}(A)$ of ${\mathfrak {M}}_{({\mathfrak {P}}^{\circ }(X),{\mathcal {F}}(\preceq ))}$ for those $A \in {\mathcal {F}}(\preceq )$ with ${\mathcal {H}}({\mathcal {S}}) = \emptyset$ for all ${\mathcal {S}} \in A$.

### Proof

By definition, the orthants of ${\mathfrak {M}}_{({\mathfrak {B}}^{\circ }(X),{\mathcal {F}}(\sqsubseteq ))}$ are in one-to-one correspondence with chains in $({\mathfrak {B}}^{\circ }(X),\sqsubseteq )$. By Corollary 1 and the definition of ${\mathcal {F}}(\preceq )$, such chains are in one-to-one correspondence with chains ${\mathfrak {C}}$ in $({\mathfrak {P}}^{\circ }(X),\preceq )$ for which ${\mathcal {H}}({\mathcal {S}}) = \emptyset$ for all ${\mathcal {S}} \in {\mathfrak {C}}$. Again by definition, the latter chains are in one-to-one correspondence with the orthants ${\mathfrak {O}}(A)$ of ${\mathfrak {M}}_{({\mathfrak {P}}^{\circ }(X),{\mathcal {F}}(\preceq ))}$ for those $A \in {\mathcal {F}}(\preceq )$ with ${\mathcal {H}}({\mathcal {S}}) = \emptyset$ for all ${\mathcal {S}} \in A$. $\square$

We remark that the characterization of geodesic paths in CAT(0)-orthant spaces in [25, Corollary 6.19] holds for equidistant-cactus space ${\mathfrak {N}}(X)$. This implies that, for any two points in ${\mathfrak {N}}(X)$ that correspond to equidistant $X$-trees, all points on the unique geodesic path between these two points also correspond to equidistant $X$-trees. In other words, $({\mathfrak {M}}_{({\mathfrak {B}}^{\circ }(X),{\mathcal {F}}(\sqsubseteq ))},D_{({\mathfrak {P}}^{\circ }(X),{\mathcal {F}}(\preceq ))})$ is a convex subspace of ${\mathfrak {N}}(X) = ({\mathfrak {M}}_{({\mathfrak {P}}^{\circ }(X),{\mathcal {F}}(\preceq ))},D_{({\mathfrak {P}}^{\circ }(X),{\mathcal {F}}(\preceq ))})$.

## Conclusion

We have introduced the space ${\mathfrak {N}}(X)$ of equidistant *X*-cactuses. By deriving an encoding for ranked *X*-cactuses, we obtained ${\mathfrak {N}}(X)$ as an orthant space and proved that it is a CAT(0)-metric space. Thus, we can compute the distance in ${\mathfrak {N}}(X)$ between any two equidistant *X*-cactuses and the unique geodesic path between them in polynomial time [25], compute approximations of the Fréchet mean and variance as well as of the median of a set of equidistant $X$-cactuses [4, 25], and a central limit theorem holds [5]. There are several directions for future research and open questions including:

- It would be interesting to count the number $\nu _n$ of isomorphism classes of binary ranked $X$-cactuses with rankings of size $|X |-1$. In view of Theorem 2, this is equivalent to counting the number of maximal chains in the graded poset $({\mathfrak {P}}(X),\preceq )$. Counting chains in certain types of posets is a well-studied problem (see e.g. [33]). The values of $\nu _n$ for $n = 1,2,3,4$ are 1, 1, 6, 72.
- It is known that the link of the origin of phylogenetic tree space as defined in [8] has the homotopy type of the wedge of spheres. It would be interesting to work out the homotopy type of the link of the origin of ${\mathfrak {N}}(X)$, and also what other properties it might enjoy (for example, is it Cohen-Macaulay as with the tree-space defined in [8]?)
- As was pointed out in [15], there is a connection between the space of circular split collections defined in [16] and a certain type of unrooted phylogenetic networks called level-1 networks. Since these unrooted level-1 networks can be regarded as unrooted *X*-cactuses, it would be interesting to investigate if there are some connections between ${\mathfrak {N}}(X)$ and the space of circular split collections.
- It would be interesting to define and understand the geometry of spaces of more complicated phylogenetic networks with arc lengths. Two obvious candidates for such an investigation are rooted level-2 networks and tree-child, time consistent networks (see [34, Chapter 10] for definitions). Moreover, one could try to relax the requirement that the phylogenetic networks are equidistant.
- How does the distance between equidistant $X$-cactuses in ${\mathfrak {N}}(X)$ compare to other distance measures between phylogenetic networks? For example, it was shown in [1] that the weighted Robinson–Foulds distance between phylogenetic trees [28] is a $\sqrt{2}$-approximation of the distance between phylogenetic trees in the tree space defined in [8].

## Data Availability

This manuscript has no associated data.

## Footnotes

1 Strictly speaking, these spaces should probably be thought of as “spaces of circular split collections”.

2 A phylogenetic $X$-cactus is also known as a rooted 2-hybrid, 1-nested phylogenetic network [29], but for simplicity, we prefer to call it a rooted $X$-cactus since if the root and directions are ignored we obtain an unrooted *X*-cactus [20].

3 Usually called *rank function* of the graded poset. We use height function instead to avoid confusion with the rankings of rooted $X$-cactuses.
